# Serum Proteomic Profiles Reflect the Stages of Myxomatous Mitral Valve Disease in Dogs

**DOI:** 10.3390/ijms24087142

**Published:** 2023-04-12

**Authors:** Dina Rešetar Maslov, Vladimir Farkaš, Ivana Rubić, Josipa Kuleš, Anđelo Beletić, Blanka Beer Ljubić, Iva Šmit, Vladimir Mrljak, Marin Torti

**Affiliations:** 1 Internal Diseases Clinic, Faculty of Veterinary Medicine, University of Zagreb, Heinzelova Street 55, 10000 Zagreb, Croatia; vfarkas@vef.hr (V.F.); irubic@vef.hr (I.R.); abeletic@vef.hr (A.B.); bljubic@vef.unizg.hr (B.B.L.); ismit@vef.unizg.hr (I.Š.); vmrljak@vef.unizg.hr (V.M.); mtorti@vef.unizg.hr (M.T.); 2 Department of Chemistry and Biochemistry, Faculty of Veterinary Medicine, University of Zagreb, Heinzelova Street 55, 10000 Zagreb, Croatia; jkules@vef.hr

**Keywords:** myxomatous mitral valve disease, proteomics, dogs, innate immune system, inflammation, echocardiography

## Abstract

Canine myxomatous mitral valve disease (MMVD) is similar to Barlow’s form of MMVD in humans. These valvulopathies are complex, with varying speeds of progression. We hypothesized that the relative abundances of serum proteins would help identify the consecutive MMVD stages and discover new disease pathways on a systemic level. To identify distinction-contributing protein panels for disease onset and progression, we compared the proteomic profiles of serum from healthy dogs and dogs with different stages of naturally occurring MMVD. Dogs were divided into experimental groups on the basis of the left-atrium-to-aorta ratio and normalized left ventricular internal dimension in diastole values. Serum was collected from healthy (N = 12) dogs, dogs diagnosed with MMVD in stages B1 (N = 13) and B2 (N = 12) (asymptomatic), and dogs diagnosed with MMVD in chronic stage C (N = 13) (symptomatic). Serum biochemistry and selected ELISAs (galectin-3, suppression of tumorigenicity, and asymmetric dimethylarginine) were performed. Liquid chromatography–mass spectrometry (LC–MS), tandem mass tag (TMT) quantitative proteomics, and statistical and bioinformatics analysis were employed. Most of the 21 serum proteins with significantly different abundances between experimental groups (*p* < 0.05, FDR ˂ 0.05) were classified as matrix metalloproteinases, protease inhibitors, scaffold/adaptor proteins, complement components, anticoagulants, cytokine, and chaperone. LC–MS TMT proteomics results obtained for haptoglobin, clusterin, and peptidase D were further validated analytically. Canine MMVD stages, including, for the first time, asymptomatic B1 and B2 stages, were successfully distinguished in dogs with the disease and healthy dogs on the basis of the relative abundances of a panel of specific serum proteins. Most proteins with significantly different abundances were involved in immune and inflammatory pathways. Their role in structural remodeling and progression of canine MMVD must be further investigated. Further research is needed to confirm the resemblance/difference with human MMVD. Proteomics data are available via ProteomeXchange with the unique dataset identifier PXD038475.

## 1. Introduction

Myxomatous mitral valve disease (MMVD) is characterized by degenerative remodeling of the extracellular matrix of the mitral valve (MV) and is considered to affect 2% to 3% of the human population [1]. In veterinary medicine, MMVD is the most prevalent heart disease and one of the most important causes of left-sided heart morbidity, congestive heart failure (CHF), and heart-associated mortality in dogs [2,3]. Both MMVD types are degenerative valvulopathies, making Barlow’s form of MMVD in humans similar to canine MMVD [1]. Due to pathophysiological similarities between human and canine MMVD, the naturally occurring canine MMVD is considered as a suitable animal model in human medicine that may facilitate the development of therapeutic strategies for MMVD in humans [4,5].

Presently, in clinical practice, dogs in MMVD chronic stage C are the most represented group. Dogs in stages B1 and B2 are diagnosed only during regular preventive examination. The major bottleneck in the clinical examination of asymptomatic canine patients remains the timely diagnosis of the transition from MMVD stage B1 into MMVD stage B2. The enhanced clinical evaluation of stage B would enable the timely introduction of medication, considering that surgical repair of valves in dogs is still expensive and not widely available. Ideally, appropriate and timely MMVD management would delay or reverse disease progression into the symptomatic phase (stage C) and prevent heart failure, as is emphasized in the 2019 American College of Veterinary Internal Medicine (ACVIM) guidelines [2]. In contrast, in human medicine, improved mitral valve surgery therapies are broadly available. However, the discovery of mechanistic-based therapeutic approaches could potentially prevent and/or slow down the advancement of early myxomatous changes into functional prolapse. The challenge in human medicine remains the discovery of molecular and cellular changes early on during disease onset. 

With improvements in research strategies and technologies, the focus has returned to better understanding canine MMVD pathophysiology [6,7,8,9,10,11,12,13] and the probable role of immune and inflammatory mechanisms in the development and progression of valvulopathies in dogs [14]. Systemic studies focused on the detection of serum proteome changes between healthy populations and dogs/humans with gradual MMVD progression are scarce [11,15], and, to the best of our knowledge, no study has compared the proteomic profiles of asymptomatic B1 and B2 stages. The application of molecular biomarkers for early diagnosis of heart disease (asymptomatic phase) is an appealing diagnostic approach. Cardiac troponin-I (cTnI), natriuretic peptides (NT-ProBNP), C-reactive protein (CRP), and soluble suppression of tumorigenicity 2 (sST2) have been used in clinical practice as human cardiovascular biomarkers to reveal the pathophysiological characteristics of heart failure, myocyte injury, ventricular wall stress, fibrosis, and cardiac remodeling [16]. The cTnI and NT-ProBNP were implemented for detection of myocardial injury and ventricular wall stress in dogs. A study by Kuleš et al. showed higher serum concentrations of these two markers in dogs diagnosed with MMVD stage C compared to healthy dogs [9] and other MMVD stages [17]. Since echocardiography and radiography (the gold diagnostic standards for canine MMVD) and veterinary cardiologists are primarily situated in larger clinics, this, as well as increased costs of examination, limit the access for some dog owners. Therefore, discovery of broadly applicable blood-based markers for detection and monitoring of MMVD progression would provide objective and facilitated disease management.

Thus, the aim of this study was to evaluate changes in quantitative proteomic profiles in serum collected from healthy dogs and those diagnosed with different stages of MMVD (stages B1, B2, and C). In this study, we focused on multiple aspects of canine MMVD, specifically, comprehensive characterization of its pathophysiology on a molecular (protein) and systemic level. The hypothesis was that we can distinguish between not only asymptomatic (stages B1 and B2) and symptomatic (stage C) dogs but also between dogs with various stages of MMVD and the healthy/control group on the basis of different abundances of serum proteins. We specially focused on the detection of potential biomarkers of asymptomatic MMVD (B stages). Due to the resemblance of canine MMVD with human MMVD, we further hypothesized that most distinction-contributing proteins will be involved in immune and inflammatory pathways crucial for canine MMVD onset and progression. These findings might contribute to (1) improved understanding of MMVD-related molecular mechanisms in disease development and progression and (2) consolidation of the dog as a relevant and valuable model of human MMVD.

In our study, tandem mass tag (TMT) quantitative proteomics and statistical and bioinformatics analysis were employed for systemic-level insight into molecular changes occurring with the onset and advancement of canine MMVD. This approach enabled the stratification of consecutive MMVD stages and healthy dogs on the basis of changes in the relative abundance of protein panels and monitoring of pathophysiological processes with disease onset and progression. Most proteins with significantly different abundances were involved in immune and inflammatory pathways.

## 2. Results

### 2.1. Patients’ Demographics and Clinical Findings

Physical examination, signalment, cardiac imaging, and medical history enabled us to classify 50 client-owned dogs into 4 experimental groups (Table 1). The patients’ genders were balanced. The dogs were between 3 and 16 years old and of different breeds, with the largest number belonging to the Cavalier King Charles Spaniel breed (Table 1). The statistical difference between the ages of control and MMVD groups was considered significant at a level of 0.05 (Kruskal–Wallis test, *p* value = 0.0255). The same was confirmed for weight of control (Kruskal–Wallis test, *p* value = 0.006). The statistical difference was highly significant for both LVIDDn (Kruskal–Wallis test, *p* < 0.0001) and LA/Ao (Kruskal–Wallis test, *p* < 0.0001). The statistical difference was shown between healthy and diseased dogs for the fractional shortening (FS, %) parameter (Kruskal–Wallis test, *p* value = 0.0002). Mitral valve E/A ratio was statistically different between MMVD stage B1 and healthy/control and other MMVD stages (Kruskal–Wallis, *p* value = 0.023). For these two echocardiographic parameters, measured values were statistically significantly higher with the progression of MMVD disease (Table 1). Additional results are in Supplementary Material A, Section S1. Supplemental on patients’ demographics and clinical findings.

### 2.2. Serum and Candidate Cardiac Biomarker Analyses

**Figure 1:**
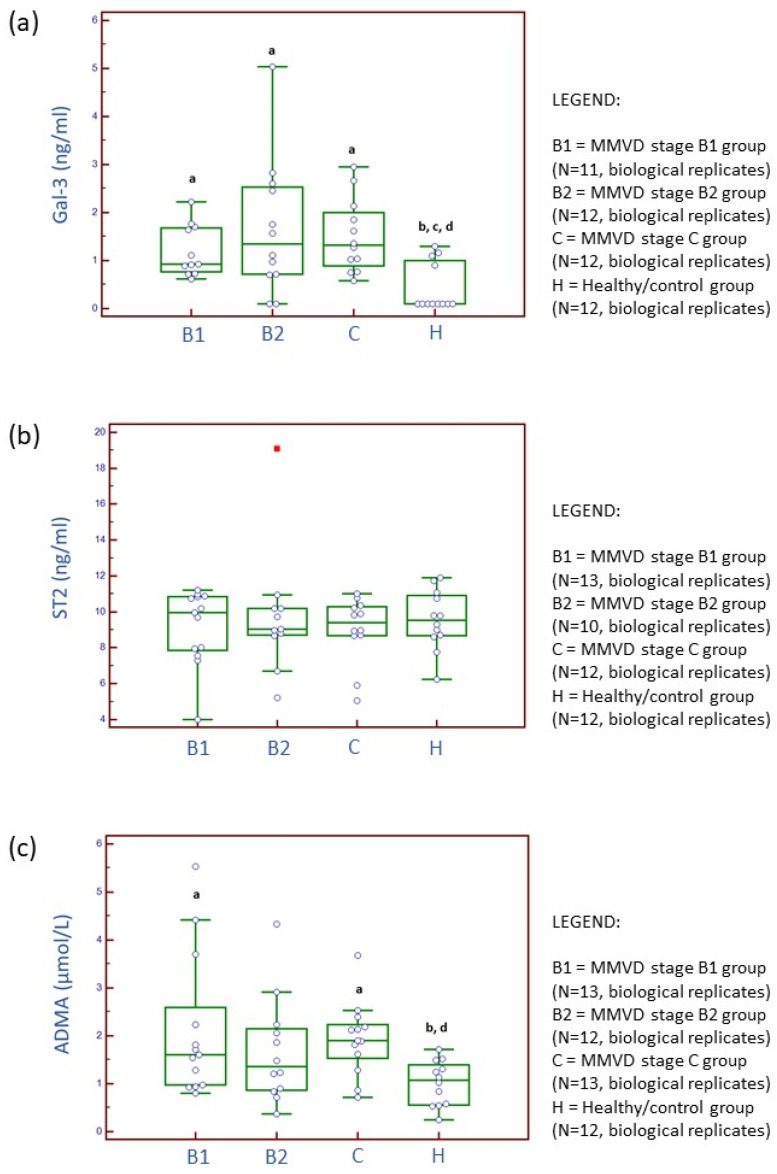
Distribution of serum concentrations for galectin-3 (Gal-3) (**a**), suppression of tumorigenicity 2 (ST2) (**b**), and asymmetric dimethylarginine (ADMA) (**c**) candidate cardiac biomarkers across three stages of naturally occurring myxomatous mitral valve disease (MMVD) and in healthy dogs. N represents the number of serum samples (biological replicates). ^a^ Statistically significantly different from control; ^b^ statistically significantly different from the MMVD stage B1; ^c^ statistically significantly different from the MMVD stage B2; ^d^ statistically significantly different from the MMVD stage C.

Table S1 (Supplementary Materials A) and Figure 1 present concentrations and activities of serum biochemical parameters and three selected cardiac biomarkers: Gal-3, ST2, and ADMA. Gal-3 concentrations were significantly higher in MMVD stage B1, B2, and C groups compared to the healthy/control group (Kruskal–Wallis test, *p* value = 0.0074). ST2 concentrations were not significantly different between experimental groups (Kruskal–Wallis test, *p* value = 0.9603). ADMA concentrations were significantly higher for MMVD stage B1 and C groups compared to the healthy/control group (Kruskal–Wallis test, *p* value = 0.0196). Further results are available in Supplementary Materials A, Section S2. Supplement on serum biochemical analysis.

### 2.3. Proteomics Results

**Figure 2:**
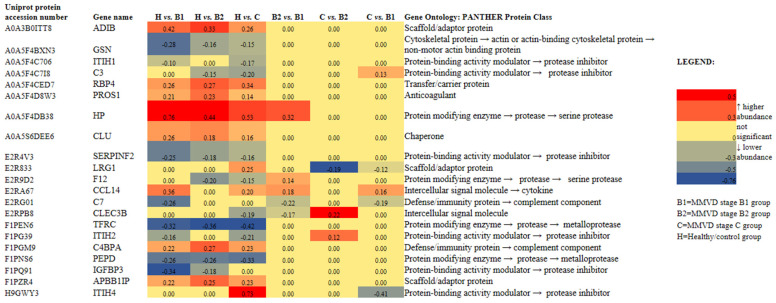
Heat map of 21 identified and quantified serum proteins with significantly different abundances (*p* < 0.05, FDR < 0.05) in dogs diagnosed with different stages (B1 (N = 13), B2 (N = 12), and C (N = 13)) of naturally occurring myxomatous mitral valve disease (MMVD) and the healthy/control group (N = 12). N represents the number of serum samples (biological replicates). A heat map was created using the log2FC values (FC = fold change) calculated for master proteins after post-hoc analysis in R. Gene ontology analysis was performed in the PANTHER classification tool with *Canis lupus familiaris* as the selected organism. The heat map was created in Microsoft Excel. ADIB = adiponectin B; GSN = actin-depolymerizing factor/gelsolin; ITIH1 = inter-alpha-trypsin inhibitor heavy chain 1; C3 = anaphylatoxin-like domain-containing protein/complement C3; RBP4 = plasma retinol-binding protein; PROS1 = vitamin K-dependent protein S; HP = haptoglobin; CLU = clusterin, SERPINF2 = serpin family F member 2; LRG1 = leucine-rich alpha-2-glycoprotein 1; F12 = coagulation factor XII; CCL14 = C-C motif chemokine; C7 = complement C7; CLEC3B = C-type lectin domain family 3 member B/tetranectin; TFRC = transferrin receptor protein 1; ITIH2 = inter-alpha-trypsin inhibitor heavy chain 2; C4BPA = complement component 4 binding protein alpha; PEPD = peptidase D; IGFBP3 = insulin-like growth factor binding protein 3; APBB1P = amyloid beta precursor protein binding family B member 1; ITIH4 = inter-alpha-trypsin inhibitor heavy chain 4.

The Kruskal–Wallis analysis identified a panel of 21 master proteins (*p* < 0.05, FDR < 0.05) with significantly different abundances in the serum of experimental groups, and post hoc showed which proteins were significantly altered between selected experimental groups (Figure 2). Further proteomics results are available in Supplementary Materials B, Section S3. Proteomics results supplement and Supplementary Materials A.

**Figure 3:**
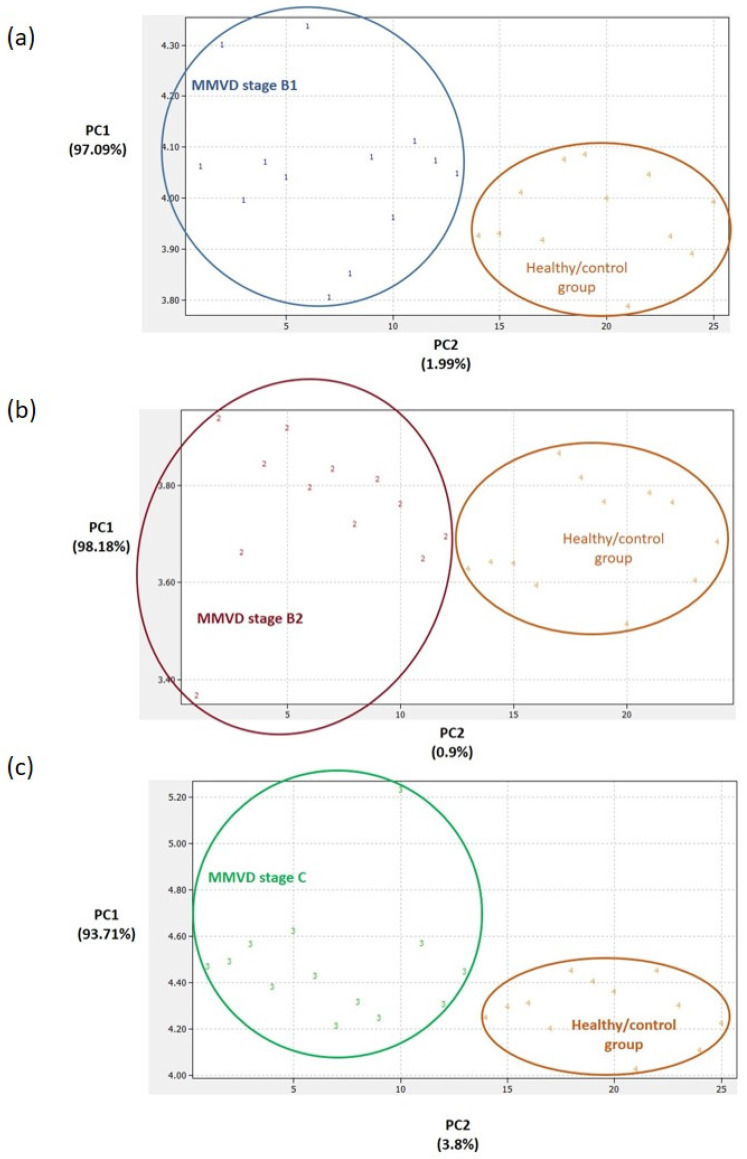
Principal component analysis (PCA) score plots. Clear differentiation between the healthy/control group and the groups with myxomatous mitral valve disease (MMVD) stages B1 (**a**), B2 (**b**), and C (**c**). In preparing the plots, relative abundance ratios of proteins with statistically different abundances (Figure 2) were used for the PCA. The PCA was performed in DataLab software. Class numbers: 1 = MMVD stage B1 (N = 13 biological replicates), 2 = MMVD stage B2 (N = 12 biological replicates), 3 = MMVD stage C (N = 13 biological replicates), and 4 = healthy/control group (N = 12 biological replicates). N represents the number of serum samples (biological replicates).

**Figure 4:**
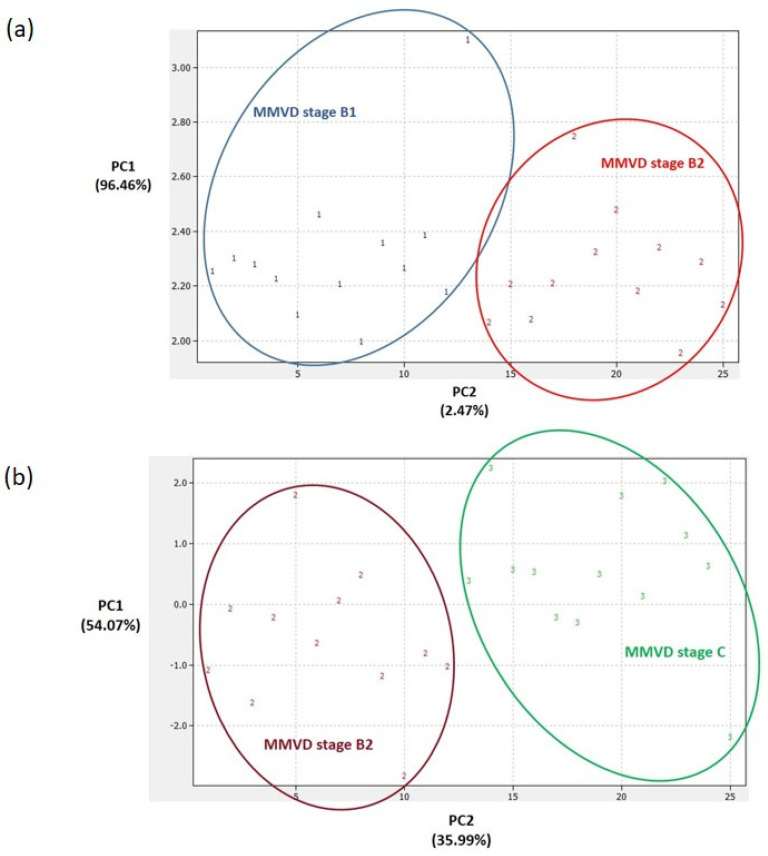
Principal component analysis (PCA) score plots. Clear differentiation between groups with consecutive myxomatous mitral valve disease (MMVD) stages B1 and B2 (**a**) and between groups with MMVD stages B2 and C (**b**). In preparing the plots, relative abundance ratios of proteins with statistically different abundances (Figure 2) were used for the PCA. The PCA was performed in DataLab software. Class numbers: 1 = MMVD stage B1 (N = 13 biological replicates), 2 = MMVD stage B2 (N = 12 biological replicates), 3 = MMVD stage C (N = 13 biological replicates), 4 = healthy/control group (N = 12 biological replicates). N represents the number of serum samples (biological replicates).

Panels of significantly different proteins (Figure 2) enabled us to clearly distinguish between the PCA score plots of (1) the groups with each MMVD stage and the healthy/control group (Figure 3), (2) the groups with MMVD stages B1 and B2 (Figure 4a), and (3) the groups with MMVD stages B2 and C (Figure 4b).

**Figure 5:**
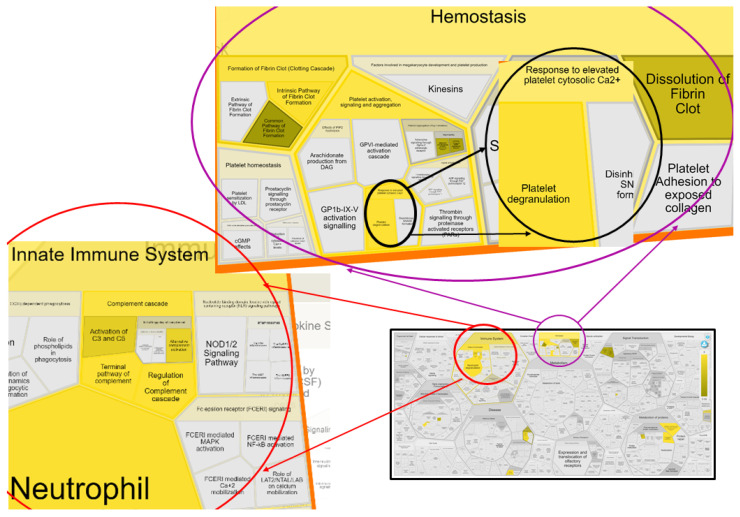
Reacfoam representation of seven biological pathways with ≥5 associated gene hits that are a part of the innate immune system and hemostasis of the circulation system. Reacfoam was exported from Reactome software following pathway enrichment analysis of serum proteins differentially abundant between groups with different MMVD stages and the healthy/control group (*p* < 0.05, FDR ˂ 0.05). Pathways that were enriched are colored yellow.

A Reactome analysis helped identify 17 out of 21 proteins in the Reactome database. Proteins represented by gene names CCL14, ADIB, ITIH1, and PEPD were not found. In all, 105 pathways were hit by at least 1 protein from the query list, while 19 pathways fulfilled the cutoff criteria (*p* < 0.05, FDR ˂ 0.05). Table 2 presents the most relevant pathways sorted by *p*-value. There were seven pathways with at least five associated gene hits (Table 2 and Figure 5). STRING recognized 20 proteins (ADIB was not found). Functional enrichment of protein–protein interaction networks can be viewed in Figure S1 in Supplementary Materials A.

### 2.4. Analytical Validation of Proteomics Results

**Figure 6:**
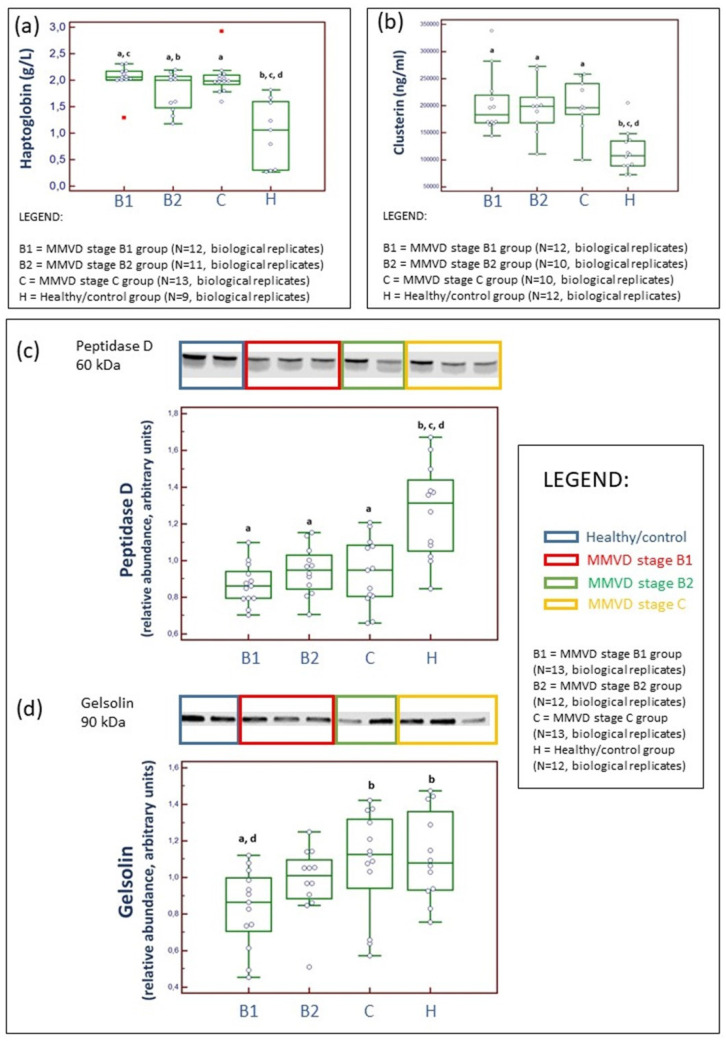
Analytical validation of proteomics results. Serum concentrations of haptoglobin (HP) measured by haptoglobin spectrophotometric assays (**a**) and clusterin assessed through an ELISA (**b**). Western blotting and example of relative density comparison of serum peptidase D (**c**) and gelsolin (**d**) proteins. N represents the number of serum samples (biological replicates). HP concentrations were significantly higher (Kruskal–Wallis test, *p* value = 0.0004) in diseased dogs compared to the healthy/control group. Moreover, HP concentrations were significantly lower in dogs with MMVD B2 stage compared to dogs with MMVD stage B1 (**a**). Clusterin concentrations were significantly higher (Kruskal–Wallis test, *p* value = 0.0082) in dogs diagnosed with MMVD compared to the healthy/control group (**b**). Peptidase D was significantly lower (Kruskal–Wallis test, *p* value = 0.0007) in diseased dogs compared to the healthy/control group (**c**). Gelsolin was significantly lower (Kruskal–Wallis test, *p* value = 0.0182) in dogs with MMVD stage B1 compared to the healthy/control group, and although values were lower for dogs with MMVD stage B2, this difference was not significant (**d**). ^a^ statistically significantly different from control; ^b^ statistically significantly different from the MMVD stage B1; ^c^ statistically significantly different from the MMVD stage B2; ^d^ statistically significantly different from the MMVD stage C.

To support differences in serum protein abundances distinguished by TMT-based proteomics and subsequent statistical analysis (Figure 2), we analytically validated selected proteins by quantitative measurements (Figure 6). Validated proteins were selected on the basis of scientific/biological importance and assay availability. Haptoglobin, clusterin, and peptidase D showed significant differences between the healthy/control group and diseased dogs (Figure 6) and showed excellent consistency and confirmation with MS-based proteomics results. Supplementary Materials A (Figure S2) provides complete, unedited Western blot membranes following the detection of two proteins.

Linearity under dilution results (linear regression equation with CV almost equal to 1 and r ˃ 0.97 for all assays) showed that the ELISA was performed accurately and with intra-assay CV below 13%.

## 3. Discussion

### 3.1. Serum Parameters and Candidate Cardiac Biomarker Analysis

Inspired by the research in human medicine, several cardiac biomarkers were targeted in the serum of dogs diagnosed with MMVD to characterize canine MMVD (Figure 1).

Protein Gal-3 is a type of soluble β-galactoside-binding lectin and has a crucial contributing role in production of collagen in the heart, following fibroblasts proliferation and phenotype change into myofibroblasts [18,19,20,21]. Gal-3 was previously detected in the extracellular matrix, cytoplasm, and nucleus. Increased concentrations of circulating and tissue Gal-3 directly correlate with macrophage migration/activation and development of myocardial fibrosis in human and murine as well as with advancement of canine MMVD. Previously, it was demonstrated that, in human and canine MMVD, the concentrations of circulating Gal-3 correlate with the amount of collagen accumulated in the heart, the incidence of CHF, and disease progression and outcome [19,22,23,24]. Our results (Figure 1) further confirm previous experimental findings and identify the Gal-3 marker as a potentially important mediator of myxomatous degenerative changes in both asymptomatic and symptomatic stages in canine MMVD. There was no significant difference in serum Gal-3 concentrations with disease progression. Therefore, this marker may not have a prognostic value but a diagnostic value for early (MMVD stage B1) detection, and diagnosis confirmation should be further carefully evaluated. Although, previously, a significantly positive correlation was found between Gal-3 and NT-proBNP and Gal-3 was shown to have predictive potential for diastolic dysfunction, Gal-3 cannot be designated either as an MMVD- or as a cardiac-specific marker. Increased values of circulating Gal-3 were previously reported for various diseases, for example, endocrine and dermatologic diseases as well as cancer [24]. Nevertheless, the inhibition of Gal-3 with modified citrus pectin, an emerging heart failure therapy for dogs, resulted in the reduced burden of reactive interstitial fibrosis in CHF and reduced and preserved ejection fraction [25]. 

In human medicine, compared to Gal-3 protein, soluble ST2 (sST2) was characterized as a superior marker for cardiac remodeling and cardiac fibrosis [26]. Although ST2 proved to be an unspecific marker, recent studies on myxomatous degeneration suggest that the cytokine interleukin-33/ST2 pathway may be amplifying extracellular matrix remodeling in mitral valves [27]. Unfortunately, studies investigating circulating ST2 concentrations in canine MMVD [17,28], including our study, have, up to now, not been successful in showing significant differences between healthy and diseased states. Further research is needed before definitive conclusions.

ADMA is continuously produced in organism as a metabolic by-product of protein methylation. It is considered a marker of oxidative stress, that is, an endogenous inhibitor of nitric oxide synthase. Previous experimental studies suggested association between nitric oxide production and MMVD pathogenesis [29], presumably due to negative effects on vasodilatation and vaso-protection. On the other hand, increased nitric oxide concentration in circulation is associated with production of a strong nitrosating agent when in contact with oxygen, which could potentially regulate transcription factors and expression of genes included in extracellular remodeling. Li et al. (2015) hypothesized, following detection of decreased ADMA and increased expression of endothelial nitric oxide synthase, that an indirect redox-based nitric oxide signaling pathway may regulate extracellular matrix changes in canine MMVD [13]. The latter study did not grade MMVD cases into stages, and severity of MMVD was not reported. Valente et al. (2021) showed a significant increase in plasmatic ADMA in dogs diagnosed with MMVD stages C and D (merged into one group) compared to the MMVD stage B1 group and the control group [30]. We hypothesize that increased serum ADMA concentrations are probably a by-product of increased protein post-translational modifications that occur early on (MMVD stage B1) and increase again with disease advancement (MMVD stage C). We are still cautious where drawing any conclusions is concerned because ADMA concentrations in circulation may also be associated with fatty liver, LDL cholesterol, obesity, and body size in dogs [29,31]. Moreover, ADMA is continuously produced in organisms as a metabolic by-product of protein methylation. In addition, a recent study reported significantly higher serum ADMA concentrations in dogs with acute pancreatitis [32]. Finally, although significant differences were shown in Gal-3 and ADMA between healthy and MMVD groups, these differences are numerically small (Figure 1). 

A discussion on serum biochemical parameters may be viewed in Supplementary Materials A, Section S2. Supplement on serum biochemical analysis.

### 3.2. Proteomic Results

To date, several studies have provided a deeper insight into the changes in serum proteomes of dogs affected by MMVD [8,9,10,11]. All these studies were performed on naturally occurring MMVD, and, so far, only one study has compared the serum profiles of consecutive MMVD stages (B2, C, and D) in comparison to stage A (healthy dogs genetically predisposed to developing MMVD) [11]. To the best of our knowledge, there is no study that has compared the proteomic profiles of asymptomatic B1 and B2 stages. Our study revealed a panel of 21 master proteins with significantly altered abundances in the serum of experimental groups (Figure 2). Most of these proteins (and protein panels) had not been discovered in previous proteomics-based research of canine MMVD, and the involvement of these proteins in biological pathways was barely tackled. Post hoc analysis revealed significantly altered proteins between different group combinations (Figure 2), and these protein panels, for the first time, enabled the distinction of canine MMVD stages in PCA (Figure 3 and Figure 4). The patient stratification based on echocardiographic measurements and experimental approach implemented in this study are the most complete so far and enabled the differentiation of asymptomatic patients (Figure 4a). The significantly altered proteins between the healthy/control group and groups with early MMVD stages (B1 and B2) as well as between groups with early MMVD stages, for example, C-C motif chemokine (CCL14) protein (Section 3.2.1), might be particularly interesting for human medicine and drug discovery research.

Most of the significantly altered proteins were classified as matrix metalloproteinases and protease inhibitors (Figure 2), whose importance in extracellular remodeling of mitral valves have previously been reported both in human and veterinary medicine. For example, in the extracellular matrix, IαI heavy chain family members interact with hyaluronan molecules, the biochemical reaction occurring during inflammation. Hyaluronan is a non-sulfated glycosaminoglycan heavily expressed on the cell surface of various tissue types. In both canine and human MMVD cases, the amount of hyaluronan increases and spreads throughout all three layers of valve tissue. Presumably, with the increase in hyaluronan in the valve, circulating ITIH1 and ITIH2 levels in MMVD reduce. In addition, a recent study revealed the direct interaction of IαI heavy chain 1 with complement C3 via a metal-ion-dependent adhesion site. The formation of the latter complex inhibits the activity of the alternative pathway C3 convertase [33]. In other words, experimental findings have suggested that IαI heavy chain 1 might be a regulator of innate immunity early on during disease onset (MMVD stage B1) and disease progression (MMVD stage C) (Figure 2). 

In this study, we have specifically focused on the potential roles of other relevant proteins: haptoglobin (Section 3.2.2), clusterin (Section 3.2.3), peptidase D (Section 3.2.4), and gelsolin (Section 3.2.5). Increased incidences of inflammatory cells, macrophages, and T cells have already been reported in human myxomatous mitral valves. On the contrary, similar studies on canine MMVD are scarce, and the studies that were finalized suggest that inflammation is unlikely to be involved in the pathological process of canine MMVD [34]. This conclusion is primarily due to poor evidence that inflammatory mediators and cells are present and active in diseased canine valves. However, a recent study has presented the up-regulation of several pro-inflammatory cytokines (tumor necrosis factor (TNF)-α, interleukin (IL)-1β, and IL-6) with the advancement of disease severity as well as the presence of CD4+FoxP3+ regulatory T cells (Treg) in affected canine valves [35]. This indicates the potential existence of a regulatory mechanism responsible for restraining the inflammatory immune response in canine MMVD. Our findings support the idea that inflammatory mechanisms contribute to pathological extracellular matrix remodeling and that the innate immune system plays a significant role in canine MMVD progression (Table 2, Section 3.2.6). These findings strengthen previously discovered similarities between not only canine and human MMVD but also between human MMVD and MMVD in pigs, mice, and sheep, species in which populations of multiple immune cells increase in number with MMVD [36]. Finally, we have addressed cell and tissue hemostasis (Section 3.2.7) and anemia of chronic disease (Section 3.2.8) as the biological pathways and mechanisms that showed up as important in canine MMVD pathology (Table 2 and Figure 5).

#### 3.2.1. C-C Motif Chemokine (CCL14) Protein-Cytokine

CCL14 is a known activator of immune cells, mainly monocytes. Monocytes and macrophages, effectors of the innate immune system, play a crucial role in the regulation of tissue homeostasis and inflammation. Therefore, their role throughout valvulogenic processes and disease development in the human population is important. In diseased valves, circulating monocytes with specific cytokine receptors presumably penetrate into valve tissue layers and differentiate into inflammatory macrophages that express factors and potentially drive disease processes in valve interstitial cells [37]. According to our results (Figure 2), the CCL14 activity is the highest early in disease onset (MMVD stage B1), increases again with the development of CHF (stage C), and is higher in the B1 stage than in the B2 stage. Interestingly, mitral valve inflammation and inflammatory proteases were reduced in the C-C chemokine receptor type 2 genetic knockout mouse model, while C-C chemokine receptor type 2 antagonist RS504393 therapy, early on or in a late phase, ameliorates myxomatous valve degeneration and valve leaflet thickness in Fbn1C1039G/+ mice [36]. A similar experimental approach may be implemented for investigating C-C motif chemokine receptor type 14^+^ immune cells. 

#### 3.2.2. Haptoglobin (HP), an Acute Phase Protein

Glycoprotein HP is one of a series of acute phase proteins (APPs) with anti-inflammatory activities. In dogs, the reference interval is between 0.3 and 3.5 mg/ml. Its relative abundance was significantly higher in groups with MMVD compared to the healthy/control group as well as in the group with MMVD B1 stage compared to the group with MMVD B2 stage (Figure 2), and LC–MS proteomics results were further analytically validated (Figure 6a). 

Serum HP levels rapidly increased with the activation of acute phase response early with disease development. This may have been a nonspecific inflammatory reaction in which hepatocytes produce APPs under cytokine stimuli following the local reaction (potential stimulation of macrophages, endothelial cells, or fibroblasts and other cell lines). However, neutrophil-derived HP, released by neutrophils in response to activation, might modulate the microenvironment, that is, reduce tissue damage by propagating anti-inflammatory activities and the clearance of any free hemoglobin that may have formed [38]. The latter is in line with our findings (Table 2). In addition, upon neutrophil activation, human neutrophils may secrete leucine-rich alpha-2-glycoprotein 1 (LRG1) protein [39]. Interestingly, such local secretion of LRG1 protein could be the result of the formation of protein–protein complexes. LRG1 protein had a higher serum abundance in the group with MMVD stage C compared to the healthy/control group and the groups with MMVD stages B1 and B2 (Figure 2), indicating its much bigger role in disease mechanisms with the development of chronic heart failure.

#### 3.2.3. Peptidase D (PEPD), a Member of the Matrixin Protein Family

PEPD is an essential enzyme in collagen metabolism and turnover [40]. The relative abundance of serum PEPD was decreased and remained relatively steady in diseased dogs in comparison to the healthy/control group (Figure 2). On the basis of the presently known facts on MMVD molecular pathobiology, a reduction in serum concentration could potentially indicate the increased activity of PEPD in the extracellular matrix of the mitral valve system. Specifically, intracellular PEPD may have a significant role in catalyzing the rate-limiting step in collagen digestion, thereby helping recycle proline for collagen synthesis and the possible renovation of the healthy matrix environment in mitral valve leaflets, chordae tendinae, and papillary muscles. To the best of our knowledge, our study is the first to have revealed the different abundances of PEPD in MMVD pathology, and these results were confirmed using two separate proteomics methods (Figure 2 and Figure 6c).

#### 3.2.4. Clusterine (CLU), a Chaperone Protein

Pathway enrichment analysis showed that CLU is involved in many MMVD-associated pathways, such as complement cascade and its regulation; platelet degranulation, activation, signaling, and aggregation; innate immune system; and hemostasis (Table 2). A cytoprotective role of CLU protein in MMVD was previously suggested [10] due to its role in clearing off cellular debris, programmed cell death, and complement-mediated cell lysis. Interestingly, in human medicine, increased levels of circulating CLU in patients with CHF were associated with worse outcome (death) [41]. Clusterin, also known in human medicine by the name apolipoprotein J, is expressed in a variety of tissues and has been detected in all human biofluids [42]. CLU aggregation was observed in serious states of physiological disturbance and in several neurodegenerative diseases, that is, states associated with advanced aging [43]. In the present study, the relative abundance of serum CLU was higher throughout MMVD progression in diseased dogs when compared to the dogs in the healthy/control group, and these results were confirmed using two separate proteomics methods (Figure 2 and Figure 6b).

#### 3.2.5. Gelsolin (GSN), a Non-Motor Actin-Binding Protein

GSN, a non-motor actin-binding protein, had lower serum abundance in diseased dogs compared to the dogs in the healthy/control group (Figure 2). Although significant changes in GSN abundance were also confirmed by Western blotting between MMVD stage B1 and stage C groups and the healthy/control group (Figure 6d), we were not able to fully confirm proteomics results obtained by the MS approach. The difference in results could be due to different sensitivities and specificities of applied analytical methods (meaning that MS has higher sensitivity and specificity compared to Western blot analysis) as well as the coexistence of different protein isoforms, which may affect ELISA and Western blot results. Previously, lower protein abundance for GSN was reported in diseased dogs (MMVD stage C) compared to controls [9]. Circulating GSN molecules are the most important catchers of actin filaments released into circulation upon cell injury. GSN is also called actin-depolymerizing factor due to its essential role in the regulation of actin filament turnover. It can play a role in innate immunity by activating macrophages and localizing inflammation. In human and canine species, abnormal α-smooth muscle actin (SMA) was detected next to heart valve interstitial cells in the mitral valve tissue of subjects with advanced MMVD stages [44,45]. Due to its ability to bind actin fibers, SMA is considered an important mediator of extracellular matrix pathological remodeling in MMVD. Interestingly, mechanical tension seems to regulate SMA expression [46]. A recent study showed that human patients with severe tissue injury had increased levels of circulating actin, and lower GSN levels were observed in patients with decreased fibrinolysis. We concluded that increased actin levels in circulation contribute to fibrinolytic shutdown due to decreased fiber resolvability [47], which can further facilitate platelet aggregation and activation [48]. The latter pathways were recognized as relevant in canine MMVD (Table 2). 

#### 3.2.6. Innate Immune System: Platelets and Complement Cascade

With disease advancement, glycosaminoglycan infiltrates the spongiosa layer and spreads into other layers, affecting elasticity and structural integrity and possibly exposing sub-endothelial collagen to circulating platelets. Collagen is considered as one of the most thrombogenic factors, and collagen–platelet interaction promotes platelet activation. Five proteins identified as significantly different in the present study (C-type lectin domain family 3 member B/tetranectin, vitamin K-dependent protein S, clusterin, inter-alpha-trypsin inhibitor heavy chain 4, and serpin family F member 2) were enriched in the following pathways: (1) platelet degranulation, (2) response to elevated platelet cytosolic Ca^2+^, and (3) platelet activation, signaling, and aggregation (Table 2). Moreover, all these proteins were also enriched in hemostasis, and several in fibrin clot formation (Table 2). Differences in serum tetranectin (CLEC3B) levels have previously been found in canine MMVD [9,11]. Due to stimulating activity on extracellular proteolysis in collagen-, fibrin-, and plasminogen-containing tissues, CLEC3B seems to play a significant role in tissue remodeling, potentially by inducing plasminogen activation. Lower abundance of circulating CLEC3B in MMVD stage C, as reported herein (Figure 2), might be a result of increased attachment and penetration into damaged heart tissue, the base of leaflets, or blood clots, contributing to the breakdown of fibrin in blood clots. Differences in CLEC3B abundance were observed between MMVD stages, indicating abundance fluctuation with disease progression (Figure 2). Moreover, the results presented herein (reduced circulating CLEC3B levels in MMVD stage C, Figure 2) agree with findings in a human heart failure study [49].

A multifunctional anticoagulant vitamin K-dependent protein S (PROS1) had a higher relative abundance in diseased dogs (Figure 2). This glycoprotein can be recognized as another key regulator of the complement cascade pathway and vascular response to damage. In humans, along with its free form, PROS1 also exists as a complex, together with the complement component 4 binding protein (C4BP), which inhibits the activity of the C3 convertase. In the present study, C4BP alpha had increased relative abundance in the serum of dogs diagnosed with MMVD (Figure 2). Increased C4BP levels may have occupied some extent of free PROS 1, hindering its activity against clot formation. In addition, there were lower levels of serpin family F member 2 (SERPINF2), a serine protease inhibitor indirectly involved in fibrinolysis through the inactivation of plasmin, in the serum of diseased dogs (MMVD stages B1, B2, and C) than in the serum of the healthy/control group. Contrary to the high association between heart failure and thromboembolic stroke risk in humans, evident clinical thromboembolism in dogs with CHF is rarely reported [50]. Recent experimental findings on hemostatic markers in dogs with CHF caused by MMVD suggest that a predisposition toward hypercoagulation, as a side effect of increased blood viscosity, may be regulated by fibrinogen and D-dimer concentrations [51]. Caivano et al. (2021) reported a clinical case with the hyperechoic mass adhering to the endocardium of the left atrial wall. Endomyocardial tears, multifocal left atrial thrombosis, and hemopericardium were confirmed during post-mortem examination [52]. There was a lower abundance of coagulation factor XII (F12), a known serine protease and a member of the coagulation cascade, in MMVD stages B2 and C (Figure 2). Reduced F12 levels can be explained as another systemic mechanism that might have contributed to the reduced formation of blood clots.

#### 3.2.7. Insulin-like Growth Factor Binding Protein 3 (IGFBP3): Cell and Tissue Hemostasis

A mutation within the insulin-like growth factor (IGF) loci was recognized as one of the crucial genetic contributors to canine MMVD pathogenesis [53]. IGF expression influences cardiac mass and function. However, the activity of free IGF in circulation is regulated via the formation of IGF complexes with transport proteins named insulin-like growth factor binding proteins (IGFBPs). The relative abundance of IGFBP3 was lower in the groups with MMVD stages B1 and B2 compared to the healthy/control group (Figure 2), which may indicate an increased rate of complex formation. In addition, IGFBP3 is known for interacting with other serum proteins, such as lactoferrin and transferrin, and binding to the extracellular matrix, for instance, fibronectin, heparin, fibrin, and collagen, which lowers its affinity for IGF. IGFBP3 stops retinal endothelial cell apoptosis by inhibiting tumor necrosis factor (TNF)-α production [54]. Therefore, the potential role of IGFBP3 in MMVD pathology is versatile and it can act independently of IGF upon association with the plasma membrane, cellular uptake, and/or its translocation into the nucleus (regulation of the transcription of cell death receptors and ligands) [55].

#### 3.2.8. Anemia of Chronic Disease

In mammals, transferrin receptor protein 1 (TRFC) is a membrane protein and the cellular receptor of the iron-binding and iron-delivering plasma protein called transferrin. Soluble transferrin receptor proteins (sTfRs) are transferrin receptor proteins cleaved from the membranes of proliferating cells; therefore, the concentration of sTfRs in serum is associated with receptor density on iron-importing cells and is considered a good measure of erythropoietic activity. In a previous study, bone-marrow-hematopoietic-derived cells were detected in human heart valves. Previous experimental findings proposed that such cells have synthetic properties of valve interstitial cells in normal valve homeostasis, such as producing collagen type I [56]. Decreased values of sTfRs in diseased dogs may indicate iron deficiency, anemia, or impaired erythropoiesis that subsequently affects the normal synthesis of collagen in MMVD. In addition, diminished levels of sTfRs in blood are associated with anemia of inflammation, also known as anemia of chronic disease, which occurs over time in humans and animals as a side effect of pathogen intrusion or the presence of inflammatory disease [57]. Specifically, chronic inflammation reduces the amount of free iron by bounding into iron–protein complexes, for example, ferritin–iron complexes, which may result in a mild drop in the hemoglobin level. The decrease in the transferrin receptor protein 1 relative abundance was more pronounced with disease progression, though the decrease was not significant (Figure 2). We further hypothesize that the activation of the innate immune system in MMVD may result in anemia of chronic disease and decreased abundance of transferrin receptor protein 1 with disease progression.

#### 3.2.9. Study Limitations

Although we were able to achieve the goals of the study, this experimental approach has several limitations. The study relies on a relatively small number of serum samples, though this is so far the proteomics-based study with the highest number of samples. Although MS-based proteomics is highly accurate, this study may be viewed as proof-of-concept (POC) and a stepping stone for future proteomics studies with a targeted MS approach and mechanistic studies. Presently, the echocardiography remains the gold standard in MMVD diagnosis. In addition, due to existence of previously reported differences between human and canine MMVD, and species in general, extrapolating between the species must be done with caution. Further research is needed to evaluate the value of canine MMVD as an animal model for human MMVD. MMVD groups were mostly composed of small- and medium-sized dogs. Future studies should focus on including larger breeds with MMVD pathology or sample groups composed of only pure-breed dogs with confirmed genetic predisposition toward the development of MMVD. Furthermore, a multi-omics approach would be much appreciated. For example, dogs diagnosed with MMVD could be included in genomics study first and, then, on the basis of genetic predisposition, divided into two experimental groups for proteomics study. Presently, this proteomics study can be viewed as POC with a successfully finalized discovery phase.

## 4. Materials and Methods

### 4.1. Study Approval, Health Assessment, Cardiac Imaging, and Serum Collection 

The Committee on the Ethics of the University of Zagreb, Faculty of Veterinary Medicine (Permit Number 640-01/14-305/16, 251-61-01/139-14-28) approved the study protocol, and dog owners provided informed consent. Client-owned dogs of different breeds, ages, and body weights admitted at the Veterinary Teaching Hospital, Internal Diseases Clinic, Faculty of Veterinary Medicine, University of Zagreb, Croatia, were enrolled. The dogs underwent complete physical examination, including auscultation of the heart and evaluation of cardiac function. All animal procedures performed were in line with the guidelines from Directive 2010/63/EU of the European Parliament on the protection of animals used for scientific purposes.

A complete physical examination was carried out, followed by transthoracic echocardiographic examination. In dogs that were arrhythmic during either the physical examination or the echocardiographic examination, a six-lead electrocardiogram (ECG) was performed using a Philips T20 Pagewriter (Koninklijke Philips N.V.). Echocardiographic examination was performed by an experienced specialist (M.T.) using a Philips Epiq CVX (Koninklijke Philips N. V.) equipped with phased-array transducers and a simultaneous single-lead electrocardiogram. The dogs were imaged from right and left parasternal positions, and standard echocardiographic two-dimensional, M-mode, and Doppler images were acquired without sedation [58].

On the basis of the results of medical history, signalment, physical examination, and echocardiography, 50 dogs were classified into 4 experimental groups: group 1, clinically healthy dogs, that is, the healthy/control group (N = 12); group 2, the MMVD stage B1 group (N = 13); group 3, the MMVD stage B2 group (N = 12); and group 4, the MMVD chronic stage C group (N = 13) (later in text addressed only as MMVD stage C group). The dogs were classified into experimental groups in agreement with the 2019 ACVIM guidelines, a staging system that defines cardiac remodeling with the usage of left-atrium-to-aorta ratio (LA/Ao) and left ventricular end-diastolic diameter normalized for body weight (LVIDdN) [2]. 

In clinically healthy dogs, neither was heart murmur detected nor was there any evidence of structural heart changes or disorders during cardiac imaging. The MMVD stage B1 and B2 experimental groups were composed of asymptomatic dogs that had a typical heart murmur but had never shown clinical signs of heart failure. Either no or minor echocardiographic evidence of cardiac remodeling and mild regurgitation were detected in the MMVD stage B1 dogs, while dogs in MMVD B2 stage showed more severe and durable mitral valve regurgitation and left atrial and ventricular enlargement. Furthermore, in stage C dogs, MMVD had severely progressed and, therefore, caused clinical signs of heart failure, along with typical respiratory distress of variable severity due to acute pulmonary edema. Dogs with MMVD stage D were not enrolled in this study.

Dogs that were included in this study had no comorbid conditions or systemic diseases other than MMVD-associated secondary diseases previously reported. Dogs with congenital heart diseases were not enrolled in this study. Healthy dogs and dogs in the MMVD B1 stage did not receive any medical therapies. Dogs in the MMVD B2 stage were receiving pimobendan (Vetmedin Vet^®^ Tablets, Boehringer Ingelheim or Cardisure^®^ Tablets, Dechra, Northwich, UK) in standard doses (0.25 mg/kg twice daily), while dogs in the MMVD C stage were additionally receiving a diuretic, either furosemide (Fursemid Tablets, Belupo, Koprivnica, Croatia) or torasemide (UpCard Tablets, Vetoquinol, Sainte-Anne, France). None of the dogs in the MMVD B2 stage or C stage were receiving angiotensin-converting-enzyme (ACE) inhibitors [59,60,61].

Venous blood samples were collected at the point of examination from both healthy dogs and dogs diagnosed with MMVD. Blood samples were allowed to clot undisturbed at room temperature (RT), and serum was collected as the supernatant after centrifugation (3500× *g* for 10 min at RT). Serum aliquots were stored at −80 °C until further analysis (Section 4.2, Section 4.3 and Section 4.4). Repeated freeze–thaw cycles were avoided.

### 4.2. Serum Biochemistry and ELISA-Based Candidate Cardiac Biomarker Analyses

Standard serum biochemistry analysis was performed as a routine procedure in the laboratory for medical biochemistry, hematology, coagulation, and cytology at the Veterinary Teaching Hospital, Internal Diseases Clinic, on an Abbott automatic analyzer (Abbott Architect c4000, Abbott, Chicago, IL, USA) by applying Abbott commercial reagents and canine-specific C-reactive protein from Gentian. In addition, serum concentrations of three candidate cardiac biomarkers were assessed: galectin-3 (Gal-3), suppression of tumorigenicity 2 (ST2, also known as interleukin 33 receptor), and asymmetric dimethylarginine (ADMA). Commercially available canine-specific ELISA kits were purchased from MyBioSource Inc. (San Diego, CA, USA, www.mybiosource.com, accessed on 14 February 2022). ADMA (catalogue number MBS2604766) and ST2 (catalogue number MBS9346824) ELISA kits were quantitative sandwich assays, while Gal-3 (catalogue number MBS740224) was a quantitative competitive type of ELISA. All ELISA reagent kits were used according to the manufacturer’s instructions. Optical density was assessed on a CLARIOStar Plus microplate reader (BMG Labtech, Ortenberg, Germany) set at two wavelengths: primary 450 nm and 540 nm (for wavelength correction). Statistical analysis was performed as explained in Section 4.5.

### 4.3. TMT-Based Proteomics and Data Analysis

#### 4.3.1. Sample Preparation and Nano-LC–MS TMT-Based Proteomics

The total protein concentration in serum was determined using a Pierce BCA protein assay kit (Thermo Scientific, Rockford, IL, USA) according to the manufacturer’s protocol. For the protein digestion workflow, 35 µg of serum proteins per sample and internal standard were brought to a final volume of 50 µl per sample by adding 0.1 M triethylammonium bicarbonate (TEAB, Thermo Scientific, Rockford, IL, USA). The internal standard sample was composed of a mixture of equal protein amounts pooled from all samples in experiment and was used as a reference for TMT data normalization. Reduction, alkylation, trypsin digestion, and TMT labeling were performed as described previously by Kuleš et al. [9]. Briefly, reduction was performed with a 200 mM dithiothreitol (DTT, Sigma-Aldrich, St. Louis, MO, USA) solution at 55 °C for 60 min, and alkylation was carried out with a 375 mM (IAA, Sigma-Aldrich, St. Louis, MO, USA) solution at RT for 30 min in the dark. Acetone was precipitated overnight at −20 °C. Protein pellets were dissolved after centrifugation in 50 μL of 0.1 M TEAB. Trypsin Gold, mass-spectrometry-grade trypsin powder, was purchased from Promega, and 1 mg/ml solution was prepared by adding 0.1 M TEAB. Trypsin was added to protein solution (trypsin-to-protein ratio of 1:35), and digestion was performed overnight at 37 °C. TMT-6-plex reagents were purchased from Thermo Scientific, Rockland, IL, USA, and dissolved according to the manufacturer’s instructions, and 19 μL of the appropriate TMT label was added to each sample. Labeling reaction was enabled for 60 min at RT and quenched by adding 5% (vol/vol) hydroxylamine (Sigma-Aldrich, St. Louis, MO, USA). Finally, five TMT-labeled serum peptide samples (separately labeled with one of the TMT labels: m/z 127, 128, 129, 130, and 131) were pooled with the internal standard sample (labeled with TMT m/z 126) into a new tube to form one 6-plex. Altogether, 10 TMT 6-plex mixtures were prepared (6-plex legend available in Supplementary Materials B). Subsequently, peptide aliquots were vacuum dried and prepared for nano-LC–MS/MS analysis.

High-resolution nano-LC–MS/MS separation and detection of TMT-labeled serum peptides was performed on the UltiMate 3000 RSLCnano system (Thermo Fisher Scientific, Germering, Germany) coupled to the Q Exactive Plus Hybrid Quadrupole-Orbitrap mass spectrometer (Thermo Fisher Scientific, Bremen, Germany). Prior to nano-LC–MS/MS analysis, vacuum-dried peptides were dissolved in a loading solvent solution (0.1% formic acid (*v*/*v*) (VWR International, Darmstadt, Germany)) in 2% acetonitrile (*v*/*v*) (Honeywell, Charlotte, North CA, USA) diluted in ultrapure water (Supelco, Bellefonte, Pennsylvania, PA, USA). Peptide trapping and desalting, nano-LC–MS/MS analysis, and Top8 data-dependent acquisition (DDA) in a positive-ion mode were performed as reported earlier [9]. Briefly, the trap column (C18 PepMap100, 5 μm, 100 A, 300 μm × 5 mm) and the analytical column (PepMap™ RSLC C18, 50 cm × 75 μm) were purchased from Thermo Fisher Scientific. Peptide trapping was performed for 12 min at a flow rate of 15 μL/min. To separate peptides on the analytical column, two mobile phases were used: mobile phase A (0.1% formic acid in water (*v*/*v*)) and mobile phase B (0.1% formic acid (*v*/*v*) in 80% acetonitrile (*v*/*v*)) diluted in ultrapure water. For peptide separation, a linear chromatographic gradient was used. For this, we followed a linear gradient program reported by Kuleš et al. [9]. In brief, linear gradient was started with an increase in mobile phase B (0.1% formic acid in 80% acetonitrile) from 5 to 45% over 120 min and then from 45 to 90% over 2 min. Then, the phase was held at 80% for 2 min and re-equilibrated at 5% for 20 min. The flow rate was 300 nL/min. 

The mass spectrometer operated in a full MS scan mode (*m/z* range from 350.0 to 1800.0). The resolution was 70,000, and the injection time was set to 120 ms. The AGC target was set to be 1 × 106 ± 2.0 Da. An isolation window was used, and dynamic exclusion was adjusted to 30 s. HCD fragmentation was carried out using collision energy (29% and 35% NCE) with a resolution of 17,500 and an AGC target of 2 × 105. Peptide precursor ions without an assigned charge state and with a charge state above +7 were not fragmented.

#### 4.3.2. MS Data Processing, Statistics, and Bioinformatics Analysis

Processing of raw data, protein identification, and relative quantification were performed in the Proteome Discoverer software (v.2.3., Thermo Fisher Scientific, Waltham, MA, USA) with an implemented SEQUEST algorithm and database search against *Canis lupus* familiars. For the database search, the reference proteome FASTA file (68,378 sequences) was downloaded from the Uniprot/SwissProt database in October 2021. The following parameters were adjusted in Proteome Discoverer software: two trypsin missed cleavage sites were allowed; precursor and fragment mass tolerances were set to be 10 ppm and 0.02 Da, respectively; and carbamidomethyl (C) was chosen as fixed peptide modification, while oxidation (M), deamidation (N and Q), and TMT 6-plex (K, peptide N-terminus) were chosen as dynamic modifications. The false discovery rate (FDR) for peptide identification was calculated with the Percolator algorithm in Proteome Discoverer. At least two unique peptides and 1% FDR were required for the extraction of confidently identified proteins. To perform relative protein quantification, a correlation was made between relative intensities of reporter ions extracted from MS/MS spectra and peptides selected for MS/MS fragmentation. The internal standard values were used to normalize relative quantification results between 6-plexes, and total peptide amount was used for normalization within one 6-plex. The data underlying this article, the mass spectrometry proteomics data, are deposited at the ProteomeXchange Consortium via the PRIDE partner repository and can be accessed using the unique dataset identifier PXD038475.

The relative quantification results exported from Proteome Discoverer were further statistically analyzed to identify differences in the relative abundance of quantified proteins (1) separately between the group in each MMVD stage (B1, B2, and C) and the healthy/control group and (2) with disease progression, firstly, between stages B1 and B2 and, secondly, between stages B2 and C. An in-house-created script was used for Kruskal–Wallis and post hoc analysis of quantified master proteins in the open-source R software (v.4.1.2) (R Core Team (2018). R: A language and environment for statistical computing. R Foundation for Statistical Computing, Vienna, Austria. Available online at https://www.R-project.org/, accessed on 10 January 2022). For uncharacterized proteins or proteins with no gene name, a further search step was performed using the Basic Local Alignment Search Tool (BLAST) (v.2.9.0) (https://www.uniprot.org/blast, accessed on 11 January 2022) [62,63] and the NIH search tool provided by the National Centre for Biotechnology Information (NCBI) (https://www.ncbi.nlm.nih.gov/, accessed on 11 January 2022). More information on the latter is provided in Supplementary Materials.

Master proteins with significantly different abundances (*p* ˂ 0.05, FDR ˂ 0.05) calculated in R were included in principal component analysis (PCA), the creation of the representative heat map, protein functional analysis, and Reactome and STRING analysis. The PCA was performed in DataLab v. 3.530 (J. Lohninger, DataLab, Epina Software Labs, Pressbaum, Austria, 2011, http://www.lohninger.com/datalab/, accessed on 14 March 2022), and the heat map was created in MS Office (Excel Professional Plus 2016). The PANTHER classification tool (v.17.0) (http://www.pantherdb.org/, accessed on 15 March 2022) [64] and STRING (v.11.5) (https://string-db.org/, accessed on 16 March 2022) were applied in protein functional analysis and the creation of functional protein association networks. Therefore, gene names of proteins were inserted into PANTHER/STRING and *Canis lupus familiaris* was selected as the query organism. The pathway over-representation analysis was performed in the REACTOME tool (Reactome database release 83, Pathway browser v. 3.7, https://reactome.org/, accessed on 17 March 2022) using the curated database of pathways and reactions in *Homo sapiens* biology [65]. A statistical (hypergeometric distribution) test was applied in the calculations of probability for each pathway, and the p-values were corrected for FDR calculated by the Benjamini–Hochberg procedure. The pathway was considered as significant when the corrected *p*-value was ˂0.05 [66,67].

### 4.4. Analytical Validation of TMT Proteomics Results

ELISA, Western blot, and haptoglobin spectrophotometric assays were performed to validate proteomics results obtained for serum samples. A canine-specific ELISA kit targeting clusterin (CLU, catalogue number: CSB-E13770c) (Cusabio, Houston, TX, USA, https://www.cusabio.com, accessed on 14 March 2022) was purchased. ELISA was performed following the manufacturer’s protocol with optical density measured on a CLARIOStar Plus microplate reader as explained in Section 2.2. Assessment of ELISA analytical performance is further described in Supplementary Materials. 

For Western blot analysis, dog-specific primary antibodies, anti-peptidase D (anti-PEPD, abin6756656) and anti-gelsolin (anti-GSN, abin6992797, also known as actin-depolymerizing factor) were purchased from Antibodies-online (Aachen, Germany, www.antibodies-online.com, accessed on 14 March 2022). Western blotting was performed as described in Supplementary Materials. 

Finally, haptoglobin concentrations were determined by an automated species-specific spectrophotometric assay [68,69] based on haptoglobin–hemoglobin binding, performed on the Abbott automatic biochemical analyzer (Abbott Architect c4000, Abbott, USA). The canine haptoglobin was used as reagent 1. Statistical analysis for data generated by all validation assays was performed as explained in Section 4.5.

### 4.5. Statistical Analysis

For numerical data obtained through routine serum biochemistry tests (Section 2.2) and validation assays (Section 2.4), the statistical evaluation included a Kruskal–Wallis test performed in MedCalc software v.12.5.0.0, with a *p*-value below 0.05 set as the cutoff for statistical significance.

## 5. Conclusions

Analysis of serum parameters, assessment of selected cardiac markers (Gal-3, ST2, and ADMA), and TMT-based serum proteomics results obtained with high-resolution MS in combination with statistical and bioinformatics approaches enabled us to comprehensively characterize canine MMVD pathobiology on molecular and systemic levels. Moreover, we were able to identify novel disease-relevant proteins (protein panels) with biomarker potential, both for early onset of disease (MMVD stage B1) and for MMVD advancement, that is, differentiation of MMVD stages B1, B2, and C. The major bottleneck in MMVD management is the timely diagnosis of MMVD stage B2. In the present study, we identified five potential serum protein markers that provided clear distinction between MMVD stages B1 and B2. Haptoglobin, clusterin, and peptidase D were analytically validated. Most proteins with significantly different abundances were involved in immune and inflammatory pathways. Their role in structural remodeling and progression of canine MMVD must be further investigated. Further research is needed to confirm the resemblance/difference with human MMVD. These proteins and pathways might be of interest for pharmaceutical companies as possible drug targets in human MMVD.

## Figures and Tables

**Table 1:** Patients’ demographics and echocardiography parameters.

Parameter	Healthy Dogs (Control)	MMVD Stage B1	MMVD Stage B2	MMVD Stage C
Demographics				
Number of dogs per group	12	13	12	13
Number of male/female dogs	4/8	7/6	4/8	8/5
Breeds (frequency)	Mixed breed (4) Belgian Shepherd (3) Dachshund (2) Labrador Retriever Beauceron Border collie	Maltese dog (4) Mixed breed (2) CKCS Havanese Poodle Shih-tzu Bichon frisé Miniature schnauzer Pekingese	CKCS (6) Maltese dog Poodle Beagle Mixed breed German spitz Miniature schnauzer	CKCS (5) Mixed breed (2) Yorkshire Terrier (2) Maltese dog Poodle Shih-tzu Bichon frisé
Age (years)				
Median (Q1–Q3)	7 (4–10) ^b, c, d^	12 (7–14) ^a^	11 (8–13) ^a^	11 (9–14) ^a^
Average ± StDev. Min Max Mode	7 ± 3 3 13 3	11 ± 3 4 15 12	10 ± 3 6 16 12	11 ± 3 6 16 11
Weight (kg)				
Median (Q1–Q3)	15.9 (8.7–26.2) ^b, c, d^	7.5 (4.7–9.9) ^a^	10.1 (5.2–11.6) ^a^	7.2 (5.1–10.2) ^a^
Average ± StDev.	19.2 ± 11.3	7.8 ± 4.1	9 ± 3.7	7.4 ± 2.9
Echocardiographic measurements				
LA/Ao				
Median (Q1–Q3)	1.3 (1.3–1.4) ^b, c, d^	1.4 (1.4–1.5) ^a, c, d^	1.78 (1.6–1.9) ^a, b, d^	2.2 (2–2.6) ^a, b, c^
Average ± StDev.	1.3 ± 0.1	1.5 ± 0.1	1.8 ± 0.17	2.26 ± 0.37
LVIDDn				
Median (Q1–Q3)	1.6 (1.5–1.7) ^c, d^	1.5 (1.5–1.7) ^c, d^	1.8 (1.6–2.0) ^a, b, d^	2.3 (2.2–2.5) ^a, b, c^
Average ± StDev.	1.6 ± 5.7	1.5 ± 0.3	1.8 ± 0.2	2.3 ± 0.2
FS%				
Median (Q1–Q3)	31.7 (28.8–35.9) ^b, c, d^	41 (39–52) ^a^	46 (42–55) ^a^	47.5 (39.3–50.8) ^a^
Average ± StDev.	32.9 ± 5.7	44 ± 8	48 ± 6	45.4 ± 6.6
MV E/A ratio				
Median (Q1–Q3)	1.2 (1.2–1.2) ^b^	0.9 (0.8–1) ^a, c, d^	1.1 (0.9–1.4) ^b^	1.7 (0.9–2.5) ^b^
Average ± StDev.	1.2 ± 0.04	0.9 ± 0.2	1.2 ± 0.3	1.8 ± 1

StDev. = standard deviation; Q1 = first quartile; Q3 = third quartile; Min = minimal value; Max = maximal value; CKCS = Cavalier King Charles Spaniel; LA/Ao = left-atrium-to-aorta ratio; LVIDDn = normalized left ventricular internal dimension in diastole; FS = fractional shortening; MV E/A = mitral valve E and A velocity. ^a^ statistically significantly different from control; ^b^ statistically significantly different from the MMVD stage B1; ^c^ statistically significantly different from the MMVD stage B2; ^d^ statistically significantly different from the MMVD stage C.

**Table 2:** Pathway enrichment analysis of serum proteins differentially abundant between groups with different MMVD stages and the healthy/control group (*p* < 0.05, FDR ˂ 0.05).

Pathway Name	Associated Gene Names	*p*-Value	FDR *
Regulation of complement cascade ^#^	C3, C4BPA, C7, PROS1, CLU	1.50 × 10^−7^	1.56 × 10^−5^
Complement cascade ^#^	C3, C4BPA, C7, PROS1, CLU	2.94 × 10^−7^	1.56 × 10^−04^
Platelet degranulation ^#^	CLEC3B, PROS1, CLU, ITIH4, SERPINF2	4.76 × 10^−6^	1.56 × 10^−04^
Response to elevated platelet cytosolic Ca^2+ #^	CLEC3B, PROS1, CLU, ITIH4, SERPINF3	6.02 × 10^−6^	1.56 × 10^−04^
Regulation of insulin-like growth factor (IGF) transport and uptake by insulin-like growth factor binding proteins (IGFBPs)	C3, ITIH2, IGFBP3	7.44 × 10^−5^	0.002
Terminal pathway of complement	C7, CLU	9.71 × 10^−5^	0.002
Platelet activation, signaling, and aggregation ^#^	CLEC3B, PROS1, CLU, ITIH4, SERPINF2	1.53 × 10^−4^	0.002
Innate immune system ^#^	HP, C4BPA, C7, LRG1, PROS1, C3, GSN, CLU	3.65 × 10^−4^	0.005
TP53 regulates transcription of death receptors and ligands	IGFBP3	4.86 × 10^−4^	0.005
Post-translational protein phosphorylation	C3, ITIH2, IGFBP3	9.59 × 10^−4^	0.009
Intrinsic pathway of fibrin clot formation	PROS1, F12	0.0010	0.009
Hemostasis ^#^	PROS1, ITIH4, F12, SERPINF2, CLEC3B, CLU	0.0030	0.020
Formation of a fibrin clot (clotting cascade)	PROS1, F12	0.0030	0.022
Retinoid metabolism disease events	RBP4	0.0040	0.025
Defective SERPING1 causes hereditary angioedema	F12	0.0050	0.037
Defective factor XII causes hereditary angioedema	F12	0.0070	0.043
Iron uptake and transport	TFRC	0.0100	0.048
TP53 regulates transcription of cell death genes	IGFBP3	0.0100	0.048
Neutrophil degranulation	HP, C3, GSN, LRG1	0.0100	0.049

* FDR = false discovery rate; ^#^ pathways with ≥ 5 associated gene hits.

## Data Availability

The mass spectrometry proteomics data have been deposited at the ProteomeXchange Consortium via the PRIDE partner repository with the unique dataset identifier PXD038475.

## Supplementary Materials

The following supporting information can be downloaded from https://www.mdpi.com/article/10.3390/ijms24087142/s1. References [70,71,72,73,74,75,76,77,78,79,80,81,82,83,84,85,86] are cited in the Supplementary Materials.

## References

[1] Oyama M.A., Elliott C., Loughran K.A., Kossar A.P., Castillero E., Levy R.J., Ferrari G. (2020). Comparative pathology of human and canine myxomatous mitral valve degeneration: 5HT and TGF-β mechanisms. Cardiovasc. Pathol..

[2] Keene B.W., Atkins C.E., Bonagura J.D., Fox P.R., Häggström J., Fuentes V.L., Oyama M.A., Rush J.E., Stepien R., Uechi M. (2019). ACVIM consensus guidelines for the diagnosis and treatment of myxomatous mitral valve disease in dogs. J. Vet. Intern. Med..

[3] Atkins C., Bonagura J., Ettinger S., Fox P., Gordon S., Haggstrom J., Hamlin R., Keene B., Luis-Fuentes V., Stepien R. (2009). Guidelines for the Diagnosis and Treatment of Canine Chronic Valvular Heart Disease. J. Vet. Intern. Med..

[4] Li Q., Larouche-Lebel É., Loughran K.A., Huh T.P., Suchodolski J.S., Oyama M.A. (2021). Metabolomics Analysis Reveals Deranged Energy Metabolism and Amino Acid Metabolic Reprogramming in Dogs with Myxomatous Mitral Valve Disease. J. Am. Heart Assoc. Cardiovasc. Cerebrovasc. Dis..

[5] Pedersen H.D., Häggström J. (2000). Mitral valve prolapse in the dog: A model of mitral valve prolapse in man. Cardiovasc. Res..

[6] Lacerda C.M.R., Disatian S., Orton E.C. (2009). Differential protein expression between normal, early-stage, and late-stage myxomatous mitral valves from dogs. Proteom. Clin. Appl..

[7] de Oliveira Martins C., Santos K.S., Ferreira F.M., Teixeira P.C., Pomerantzeff P.M.A., Brandão C.M.A., Sampaio R.O., Spina G.S., Kalil J., Guilherme L. (2014). Distinct mitral valve proteomic profiles in rheumatic heart disease and myxomatous degeneration. Clin. Med. Insights Cardiol..

[8] Locatelli C., Piras C., Riscazzi G., Alloggio I., Spalla I., Soggiu A., Greco V., Bonizzi L., Roncada P. (2017). Serum proteomic profiles in CKCS with Mitral valve disease. BMC Vet. Res..

[9] Kuleš J., Bilić P., Horvatić A., Kovačević A., Guillemin N., Ljubić B.B., Galan A., Jović I., Torti M. (2020). Serum proteome profiling in canine chronic valve disease using a TMT-based quantitative proteomics approach. J. Proteom..

[10] Levent P., Kocaturk M., Akgun E., Saril A., Cevik O., Baykal A.T., Tanaka R., Ceron J.J., Yilmaz Z. (2020). Platelet proteome changes in dogs with congestive heart failure. BMC Vet. Res..

[11] Saril A., Kocaturk M., Shimada K., Uemura A., Akgün E., Levent P., Baykal A.T., Prieto A.M., Agudelo C.F., Tanaka R. (2022). Serum Proteomic Changes in Dogs with Different Stages of Chronic Heart Failure. Animals.

[12] Wilshaw J., Boswood A., Chang Y.M., Sands C.J., Camuzeaux S., Lewis M.R., Xia D., Connolly D.J. (2022). Evidence of altered fatty acid metabolism in dogs with naturally occurring valvular heart disease and congestive heart failure. Metabolomics.

[13] Li Q., Freeman L.M., Rush J.E., Huggins G.S., Kennedy A.D., Labuda J.A., Laflamme D.P., Hannah S.S. (2015). Veterinary Medicine and Multi-Omics Research for Future Nutrition Targets: Metabolomics and Transcriptomics of the Common Degenerative Mitral Valve Disease in Dogs. OMICS.

[14] Libby P., Mallat Z., Weyand C. (2021). Immune and inflammatory mechanisms mediate cardiovascular diseases from head to toe. Cardiovasc. Res..

[15] Deroyer C., Magne J., Moonen M., Le Goff C., Dupont L., Hulin A., Radermecker M., Colige A., Cavalier E., Kolh P. (2015). New biomarkers for primary mitral regurgitation. Clin. Proteom..

[16] Hara A., Niwa M., Kanayama T., Noguchi K., Niwa A., Matsuo M., Kuroda T., Hatano Y., Okada H., Tomita H. (2020). Galectin-3: A Potential Prognostic and Diagnostic Marker for Heart Disease and Detection of Early Stage Pathology. Biomolecules.

[17] Klein S., Nolte I., Granados-Soler J.L., Lietz P., Sehn M., Raue J.F., Rohn K., Packeiser E.M., Bach J.P. (2022). Evaluation of new and old biomarkers in dogs with degenerative mitral valve disease. BMC Vet. Res..

[18] De Boer R.A., Voors A.A., Muntendam P., Van Gilst W.H., Van Veldhuisen D.J. (2009). Galectin-3: A novel mediator of heart failure development and progression. Eur. J. Heart Fail..

[19] Ho J.E., Liu C., Lyass A., Courchesne P., Pencina M.J., Vasan R.S., Larson M.G., Levy D. (2012). Galectin-3, a Marker of Cardiac Fibrosis, Predicts Incident Heart Failure in the Community. J. Am. Coll. Cardiol..

[20] Lok D.J.A., Van Der Meer P., De La Porte P.W.B.A., Lipsic E., Van Wijngaarden J., Hillege H.L., Van Veldhuisen D.J. (2010). Prognostic value of galectin-3, a novel marker of fibrosis, in patients with chronic heart failure: Data from the DEAL-HF study. Clin. Res. Cardiol..

[21] Sakarin S., Rungsipipat A., Surachetpong S.D. (2016). Galectin-3 in cardiac muscle and circulation of dogs with degenerative mitral valve disease. J. Vet. Cardiol..

[22] De Boer R.A., Lok D.J.A., Jaarsma T., Van Der Meer P., Voors A.A., Hillege H.L., Van Veldhuisen D.J. (2011). Predictive value of plasma galectin-3 levels in heart failure with reduced and preserved ejection fraction. Ann. Med..

[23] Lopez-Andrés N., Rossignol P., Iraqi W., Fay R., Nuée J., Ghio S., Cleland J.G.F., Zannad F., Lacolley P. (2012). Association of galectin-3 and fibrosis markers with long-term cardiovascular outcomes in patients with heart failure, left ventricular dysfunction, and dyssynchrony: Insights from the CARE-HF (Cardiac Resynchronization in Heart Failure) trial. Eur. J. Heart Fail..

[24] Lee G.W., Kang M.H., Ro WBin Song D.W., Park H.M. (2021). Circulating Galectin-3 Evaluation in Dogs with Cardiac and Non-cardiac Diseases. Front. Vet. Sci..

[25] Sabbah H.N., Singh-Gupta V., Ramesh C.G. (2018). Abstract 12472: Inhibition of Galectin-3 Reverses Reactive Interstitial Fibrosis in Left Ventricular Myocardium of Dogs with Chronic Heart Failure. Circulation.

[26] Patric B., Camille A., Mauro I., Angelika H.-L., Tobias B., Christian M., Alan M., Frank R. (2019). Soluble ST2—A new biomarker in heart failure. Cardiovasc. Med..

[27] Garcia-Pena A., Ibarrola J., Navarro A., Sadaba A., Tiraplegui C., Garaikoetxea M., Arrieta V., Matilla L., Fernández-Celis A. (2021). Activation of the Interleukin-33/ST2 Pathway Exerts Deleterious Effects in Myxomatous Mitral Valve Disease. Int. J. Mol. Sci..

[28] Kim J.K., Park J.S., Seo K.W., Song K.H. (2018). Evaluation of ST2 and NT-proBNP as Cardiac Biomarkers in Dogs with Chronic Mitral Valve Disease. J. Vet. Clin..

[29] Pedersen L.G., Tarnow I., Olsen L.H., Teerlink T., Pedersen H.D. (2006). Body size, but neither age nor asymptomatic mitral regurgitation, influences plasma concentrations of dimethylarginines in dogs. Res. Vet. Sci..

[30] Valente C., Guglielmini C., Baron Toaldo M., Romito G., Artusi C., Brugnolo L., Contiero B., Poser H. (2021). Plasmatic Dimethylarginines in Dogs with Myxomatous Mitral Valve Disease. Front. Vet. Sci..

[31] Ci’Han H., Tural M. (2019). Assessment of asymmetric dimethyl arginine, cardiac troponin I, thyroxine, cholesterol, and triglyceride levels in obese dogs and dogs with normal body condition. Turk. J. Vet. Anim. Sci..

[32] Gori E., Pierini A., Lippi I., Meucci V., Perondi F., Marchetti V. (2020). Evaluation of asymmetric dimethylarginine as an inflammatory and prognostic marker in dogs with acute pancreatitis. J. Vet. Intern. Med..

[33] Briggs D.C., Langford-Smith A.W.W., Birchenough H.L., Jowitt T.A., Kielty C.M., Enghild J.J., Baldock C., Milner C.M. (2020). Inter-α-inhibitor heavy chain-1 has an integrin-like 3D structure mediating immune regulatory activities and matrix stabilization during ovulation. J Biol Chem..

[34] Aupperle H., Disatian S. (2012). Pathology, protein expression and signaling in myxomatous mitral valve degeneration: Comparison of dogs and humans. J. Vet. Cardiol..

[35] Piantedosi D., Musco N., Palatucci A.T., Carriero F., Rubino V., Pizzo F., Nasir S., Molinaro G., Ruggiero G., Terrazzano G. (2022). Pro-Inflammatory and Immunological Profile of Dogs with Myxomatous Mitral Valve Disease. Vet. Sci..

[36] Xu N., Yutzey K.E. (2022). Therapeutic CCR2 Blockade Prevents Inflammation and Alleviates Myxomatous Valve Disease in Marfan Syndrome. JACC Basic Transl. Sci..

[37] Sridhar S., Pham D.H., Gee T.W., Hua J., Butcher J.T. (2018). Monocytes and macrophages in heart valves: Uninvited guests or critical performers?. Curr. Opin. Biomed. Eng..

[38] Theilgaard-Mönch K., Jacobsen L.C., Nielsen M.J., Rasmussen T., Udby L., Gharib M., Arkwright P.D., Gombart A.F., Calafat J., Moestrup S.K. (2006). Haptoglobin is synthesized during granulocyte differentiation, stored in specific granules, and released by neutrophils in response to activation. Blood.

[39] Druhan L.J., Lance A., Li S., Price A.E., Emerson J.T., Baxter S.A., Gerber J.M., Avalos B.R. (2017). Leucine Rich α-2 Glycoprotein: A Novel Neutrophil Granule Protein and Modulator of Myelopoiesis. PLoS ONE.

[40] Eni-Aganga I., Lanaghan Z.M., Balasubramaniam M., Dash C., Pandhare J. (2021). PROLIDASE: A Review from Discovery to its Role in Health and Disease. Front. Mol. Biosci..

[41] Turkieh A., Fertin M., Bouvet M., Mulder P., Drobecq H., Lemesle G., Lamblin N., De Groote P., Porouchani S., Chwastyniak M. (2018). Expression and Implication of Clusterin in Left Ventricular Remodeling After Myocardial Infarction. Circ. Heart Fail..

[42] Trougakos I.P., Gonos E.S. (2002). Clusterin/Apolipoprotein J in human aging and cancer. Int. J. Biochem. Cell Biol..

[43] Yuste-Checa P., Bracher A., Hartl F.U. (2022). The chaperone Clusterin in neurodegeneration−friend or foe?. BioEssays.

[44] Rabkin E., Aikawa M., Stone J.R., Fukumoto Y., Libby P., Schoen F.J. (2001). Activated interstitial myofibroblasts express catabolic enzymes and mediate matrix remodeling in myxomatous heart valves. Circulation.

[45] Darke P.G. (1987). Valvular incompetence in cavalier King Charles spaniels. Vet. Rec..

[46] Dye B.K., Butler C., Lincoln J. (2020). Smooth Muscle α-Actin Expression in Mitral Valve Interstitial Cells is Important for Mediating Extracellular Matrix Remodeling. J. Cardiovasc. Dev. Dis..

[47] Coleman J.R., Moore E.E., Freeman K., Grubinger N.D., Hennig G.W., Cohen M.J., Samuels J.M., Hansen K. (2020). Actin is associated with tissue injury in trauma patients and produces a hypercoagulable profile in vitro. J. Trauma Acute Care Surg..

[48] Liu Y., Yin H., Jiang Y., Xue M., Guo C., Shi D., Chen K. (2013). Correlation between Platelet Gelsolin and Platelet Activation Level in Acute Myocardial Infarction Rats and Intervention Effect of Effective Components of Chuanxiong Rhizome and Red Peony Root. Evid. Based Complement. Alternat. Med..

[49] McDonald K., Glezeva N., Collier P., O’Reilly J., O’Connell E., Tea I., Russell-Hallinan A., Tonry C., Pennington S. (2020). Tetranectin, a potential novel diagnostic biomarker of heart failure, is expressed within the myocardium and associates with cardiac fibrosis. Sci. Rep..

[50] Tarnow I., Falk T., Tidholm A., Martinussen T., Jensen A.L., Olsen L.H., Pedersen H.D., Kristensen A.T. (2007). Hemostatic Biomarkers in Dogs with Chronic Congestive Heart Failure. J. Vet. Intern. Med..

[51] Prihirunkit K., Sastravaha A., Lekcharoensuk C., Chanloinapha P. (2014). Hemostatic Markers in Congestive Heart Failure Dogs with Mitral Valve Disease. J. Vet. Med..

[52] Caivano D., Marchesi M.C., Birettoni F., Lepri E., Porciello F. (2021). Left Atrial Mural Thrombosis and Hemopericardium in a Dog with Myxomatous Mitral Valve Disease. Vet. Sci..

[53] Burchell R.K., Schoeman J.P. (2014). Advances in the understanding of the pathogenesis, progression and diagnosis of myxomatous mitral valve disease in dogs. J. S. Afr. Vet. Assoc..

[54] Zhang Q., Steinle J.J. (2014). IGFBP-3 inhibits TNF-α production and TNFR-2 signaling to protect against Retinal Endothelial Cell Apoptosis. Microvasc. Res..

[55] Varma Shrivastav S., Bhardwaj A., Pathak K.A., Shrivastav A. (2020). Insulin-Like Growth Factor Binding Protein-3 (IGFBP-3): Unraveling the Role in Mediating IGF-Independent Effects within the Cell. Front. Cell Dev. Biol..

[56] Visconti R.P., Ebihara Y., LaRue A.C., Fleming P.A., McQuinn T.C., Masuya M., Minamiguchi H., Markwald R.R., Ogawa M., Drake C.J. (2006). An In Vivo Analysis of Hematopoietic Stem Cell Potential. Circ. Res..

[57] Chikazawa S., Dunning M.D. (2016). A review of anaemia of inflammatory disease in dogs and cats. J. Small Anim. Pract..

[58] Thomas W.P., Gaber C.E., Jacobs G.J., Kaplan P.M., Lombard C.W., Vet M., Moise N.S., Moses B.L. (1993). Recommendations for standards in transthoracic two-dimensional echocardiography in the dog and cat. Echocardiography Committee of the Specialty of Cardiology, American College of Veterinary Internal Medicine. J. Vet. Intern. Med..

[59] Kvart C., Häggström J., Pedersen H.D., Hansson K., Eriksson A., Järvinen A.K., Tidholm A., Bsenko K., Ahlgren E., Ilves M. (2002). Efficacy of Enalapril for Prevention of Congestive Heart Failure in Dogs with Myxomatous Valve Disease and Asymptomatic Mitral Regurgitation. J. Vet. Intern. Med..

[60] Atkins C.E., Keene B.W., Brown W.A., Coats J.R., Crawford M.A., DeFrancesco T.C., Edwards N.J., Fox P.R., Lehmkuhl L.B., Luethy M.W. (2007). Results of the veterinary enalapril trial to prove reduction in onset of heart failure in dogs chronically treated with enalapril alone for compensated, naturally occurring mitral valve insufficiency. J. Am. Vet. Med. Assoc.

[61] Wess G., Kresken J.G., Wendt R., Gaugele J., Killich M., Keller L., Simak J., Holler P., Bauer A. (2020). Efficacy of adding ramipril (VAsotop) to the combination of furosemide (Lasix) and pimobendan (VEtmedin) in dogs with mitral valve degeneration: The VALVE trial. J. Vet. Intern. Med..

[62] Altschul S.F., Madden T.L., Schäffer A.A., Zhang J., Zhang Z., Miller W., Lipman D.J. (1997). Gapped BLAST and PSI-BLAST: A new generation of protein database search programs. Nucleic Acids Res..

[63] Schäffer A.A., Aravind L., Madden T.L., Shavirin S., Spouge J.L., Wolf Y.I., Koonin E.V., Altschul S.F. (2001). Improving the accuracy of PSI-BLAST protein database searches with composition-based statistics and other refinements. Nucleic Acids Res..

[64] Mi H., Ebert D., Muruganujan A., Mills C., Albou L.P., Mushayamaha T., Thomas P.D. (2021). PANTHER version 16: A revised family classification, tree-based classification tool, enhancer regions and extensive API. Nucleic Acids Res..

[65] Gillespie M., Jassal B., Stephan R., Milacic M., Rothfels K., Senff-Ribeiro A., Griss J., Sevilla C., Matthews L. (2022). The reactome pathway knowledgebase 2022. Nucleic Acids Res..

[66] Fabregat A., Sidiropoulos K., Viteri G., Forner O., Marin-Garcia P., Arnau V., D’Eustachio P., Stein L., Hermjakob H. (2017). Reactome pathway analysis: A high-performance in-memory approach. BMC Bioinform..

[67] Fabregat A., Sidiropoulos K., Garapati P., Gillespie M., Hausmann K., Haw R., Jassal B., Jupe S., Korninger F. (2016). The Reactome pathway Knowledgebase. Nucleic Acids Res..

[68] Eckersall P.D., Duthie S., Safi S., Moffatt D., Horadagoda N.U., Doyle S., Parton R., Bennett D., Fitzpatrick J.L. (1999). An automated biochemical assay for haptoglobin: Prevention of interference from albumin. Comp. Haematol. Int..

[69] Brady N., O’Reilly E.L., McComb C., Macrae A.I., Eckersall P.D. (2019). An immunoturbidimetric assay for bovine haptoglobin. Comp. Clin. Path..

[70] Kjelgaard-Hansen M., Jacobsen S. (2011). Assay Validation and Diagnostic Applications of Major Acute-phase Protein Testing in Companion Animals. Clin. Lab. Med..

[71] Goggs R., Myers M., De Rosa S., Zager E., Fletcher D.J. (2017). Chloride:Sodium Ratio May Accurately Predict Corrected Chloride Disorders and the Presence of Unmeasured Anions in Dogs and Cats. Front. Vet. Sci..

[72] Ortega O., Rodriguez I., Hinostroza J., Laso N., Callejas R., Gallar P., Mon C., Herrero J.C., Ortiz M., Oliet A. (2011). Serum Alkaline Phosphatase Levels and Left Ventricular Diastolic Dysfunction in Patients with Advanced Chronic Kidney Disease. Nephron Extra.

[73] Morningstar J.E., Gensemer C., Moore R., Fulmer D., Beck T.C., Wang C., Moore K., Guo L., Sieg F., Nagata Y. (2021). Mitral Valve Prolapse Induces Regionalized Myocardial Fibrosis. J. Am. Heart Assoc..

[74] Urfer S.R., Kaeberlein T.L., Mailheau S., Bergman P.J., Creevy K.E., Promislow D.E.L., Kaeberlein M. (2017). Asymptomatic heart valve dysfunction in healthy middle-aged companion dogs and its implications for cardiac aging. GeroScience.

[75] Wang Y., Liu M.-J., Yang H.-M., Ma C.-Y., Jia P.-Y., Jia D.-L., Hou A.-J. (2018). Association between increased serum alkaline phosphatase and the coronary slow flow phenomenon. BMC Cardiovasc Disord..

[76] Zimmerman K.L., Panciera D.L., Hoeschele I., Monroe W.E., Todd S.M., Werre S.R., Leroith T., Fecteau K., Lake B.B. (2018). Adrenocortical Challenge Response and Genomic Analyses in Scottish Terriers With Increased Alkaline Phosphate Activity. Front. Vet. Sci..

[77] Aktas M., Auguste D., Lefebvre H.P., Toutain P.L., Braun J.P. (1993). Creatine kinase in the dog: A review. Vet. Res. Commun..

[78] Bakirel U., Gunes S. (2009). Value of cardiac markers in dogs with chronic mitral valve disease. Acta Vet..

[79] Adin D., Atkins C., Londoño L., Del Nero B. (2020). Correction of serum chloride concentration in dogs with congestive heart failure. J. Vet. Intern. Med..

[80] Adin D., Kurtz K., Atkins C., Papich M.G., Vaden S. (2019). Role of electrolyte concentrations and renin-angiotensin-aldosterone activation in the staging of canine heart disease. J. Vet. Intern. Med..

[81] Banner N.R., Bonser R.S., Clark A.L., Clark S., Cowburn P.J., Gardner R.S., Kalra P.R., McDonagh T., Rogers C.A., Swan L. (2011). UK guidelines for referral and assessment of adults for heart transplantation. Heart.

[82] Christopoulou E.C., Filippatos T.D., Megapanou E., Elisaf M.S., Liamis G. (2017). Phosphate imbalance in patients with heart failure. Heart Fail. Rev..

[83] Kendrick J., Kestenbaum B., Chonchol M. (2011). Phosphate and Cardiovascular Disease. Adv. Chronic Kidney Dis..

[84] Voelkl J., Egli-Spichtig D., Alesutan I., Wagner C.A. (2021). Inflammation: A putative link between phosphate metabolism and cardiovascular disease. Clin. Sci..

[85] Szczepankiewicz B., Pasławska U., Siwińska N., Plens K., Pasławski R. (2021). Evaluation of the diagnostic value of the renal resistive index as a marker of the subclinical development of cardiorenal syndrome in MMVD dogs. J. Renin-Angiotensin-Aldosterone Syst..

[86] Lan Q., Zheng L., Zhou X., Wu H., Buys N., Liu Z., Sun J., Fan H. (2021). The Value of Blood Urea Nitrogen in the Prediction of Risks of Cardiovascular Disease in an Older Population. Front. Cardiovasc. Med..

