# Supplementary material for: Serum Proteomic Profiles Reflect the Stages of Myxomatous Mitral Valve Disease in Dogs
Source: Int J Mol Sci. 2023 Apr 12;24(8):7142. doi: 10.3390/ijms24087142 (PMC10138901; doi:10.3390/ijms24087142)
Supplement: Supplementary file 1 [file ijms-24-07142-s001.zip › ijms-2269058-Supplementary Materials A.pdf]

Supplementary Material A

Serum Proteomic Profiles Reflect the Stages of Myxomatous Mitral Valve Disease in Dogs

Dina Rešetar Maslov [1,*], Vladimir Farkaš [1], Ivana Rubić [1], Josipa Kuleš [2], Anđelo Beletić [1], Blanka Beer Ljubić [1], Iva Šmit [1], Vladimir Mrljak [1] and Marin Torti [1]

[1] Internal Diseases Clinic, Faculty of Veterinary Medicine, University of Zagreb, Heinzelova Street 55, 10000 Zagreb, Croatia
[2] Department of Chemistry and Biochemistry, Faculty of Veterinary Medicine, University of Zagreb, Heinzelova Street 55, 10000 Zagreb, Croatia
* Correspondence: drmaslov@vef.hr; Tel.: +38-52-390-329

Supplemental methods

Bioinformatics analysis: For uncharacterized proteins, the Basic Local Alignment Search Tool (BLAST) (v.2.9.0) (https://www.uniprot.org/blast) enabled finding of other proteins in the protein sequence database with highest sequence similarity to the query protein (1,2). For proteins with provided locus but no gene name in the Proteome Discoverer export, a gene name search was performed through the NIH search tool provided by the National Centre for Biotechnology Information (NCBI) (https://www.ncbi.nlm.nih.gov/). Uncharacterized protein with Uniprot accession number E2R9D2 (gene name F12) showed 99.5% sequence similarity with coagulation factor XII (Uniprot accession number A0A8I3NR05, gene name F12) via BLAST search. Gene search for query LOC484960 (Uniprot accession number A0A5F4C7I8) performed with the NIH NCBI search tool provided additional gene description for this protein in *Canis lupus familiaris* database, which pointed that this protein is complement C3 (gene name C3). For peptidase S1 domain containing protein (Uniprot accession number A0A5F4DB38) BLAST search showed 92.4% sequence similarity with haptoglobin protein (Uniprot accession number P19006, gene name HP). Finally, the NIH NCBI search for query LOC479668 in *Canis lupus familiaris* database revealed that this is a haptoglobin-like protein. Consequently, gene names F12, C3 and HP were used in further analysis and interpretation of proteomics data.

Analytical validation of proteomics results: To evaluate analytical performance for performed ELISA assay, assay precision and accuracy was calculated as previously reported (3,4). Briefly, to evaluate accuracy serum was serially diluted with diluent suggested by ELISA kit producer and analysis was performed. Linearity under dilution curve was used for indirect assessment of assay accuracy. Intra-assay coefficient of variation (CV) was calculated from results obtained for repeated analysis of one serum sample during the same ELISA experiment.

For Western blotting, firstly SDS-PAGE electrophoresis was performed. Thus, serum samples (25 µg of proteins per sample in 5 µl dH2O) were boiled for 8 min at 95 °C in 2x Laemmli sample buffer (4.75 µl) and ß-mercaptoethanol (0.25 µl) and loaded on 12-well Mini-Protean® TGX™ precast polyacrylamide (4-15%) gels (Bio-Rad, Hercules, USA). As a marker, LI-COR WesternSure® Pre-stained Chemiluminescent Protein Ladder was used. After SDS-PAGE electrophoresis (5 min at 50 V then 60 min at 120 V), proteins were transferred to nitrocellulose membranes on Trans-Blot Turbo Transfer System (Bio-Rad, Hercules, USA) using a pre-programmed three-minute protocol for Mini-Protean® TGX™ gels. To determine the total amount of transferred proteins in every line, the membranes were stained with Ponceau S protein stain and imaged on Odyssey Fc imager (LI-COR, Bad Homburg, Germany) using a 600 nm channel. Membranes were washed 3 times for 5 min with fresh PBS buffer and then blocked for 1h at RT on orbital shaker in blocking buffer 10X (Abcam, Cambridge, UK) diluted 1:10 with dH2O. Subsequently, the membranes were incubated overnight at 4 °C with primary antibodies in blocking buffer: 1) anti-gelsolin solution (2 µg/ml) and 2) anti-PEPD solution (1 µg/ml). Next day, membranes were washed 3 x 5 min with TBST buffer and incubated with secondary antibodies for 1h at RT with orbital shaking. For anti-gelsolin, m-IgGκ BP-HRP (Santa Cruz Biotechnology Inc, sc-516102) and anti-PEPD, mouse monoclonal anti-rabbit IgG light chain HRP (Abcam ab99697) were used as secondary antibodies. After incubation, membranes were washed 4 times per 5 min with TBST and proteins were visualized by chemiluminescence using HRP chemiluminescence blotting substrate (Radiance Plus, Azure Biosystems, USA) on Odyssey Fc imager (LI-COR, Bad Homburg, Germany) using „chemi“ and 700 nm channel. Obtained images were quantified using ImageJ software (National Institutes of Health). The abundance of the proteins of interest was normalized to the total amount of protein in each lane determined with Ponceau S.

Section S1. Supplemental on patient's demographics and clinical findings

The healthy/control group (N=12; 24%) consisted of four males (33%) and eight females (67%) between three (Min) and 13 (Max) years old and median equal to seven. This group was composed of dogs of five different breeds, and four dogs were of mixed breed (Table 1). The three most frequent breeds in the control/healthy group were mixed breed (N=4; 33%), Belgian Shepherd (N=3; 25%) and Dachshund (N=2; 17%). Dogs in the control group were healthy dogs and they did not show clinical or physical changes or symptoms at the time of examination.

The three most frequent breeds of dogs diagnosed with MMVD, regardless of the MMVD stage, were CKCS (N=12; 32%), Maltese dog (N=6; 16%) and mixed breed (N=5; 13%). The majority of dogs diagnosed with MMVD were of small and medium-sized breeds. Patients' gender was balanced, an equal number of male (N=19; 50%) and female (N= 19; 50%) dogs were included in the study. The youngest dog diagnosed with MMVD was a four-year-old CKCS male, diagnosed with MMVD stage B1. The oldest dogs were a female Poodle (MMVD stage B2) and a female of mix breed (MMVD stage C). Both were 16 years old. Of 38 diagnosed dogs with MMVD, 13 (34%) dogs were younger than 10 years. Seven (54%) of these 13 dogs were of CKCS breed. Furthermore, five (13%) dogs were ≤ 6 years old.

The MMVD stage B1 group (N=13; 26%) was composed of seven males and eight females between four (Min) and 15 (Max) years, with an average of 11 (±3), a mode value of 12 (Table 1). In addition, the youngest dog diagnosed with the MMVD stage B1 was a four-year-old CKCS and the oldest were two female dogs, first of mixed breed and the second was Maltese, both were 15 years old. The two most frequently included dog breeds in MMVD stage B1 were Maltese dog (N=4; 30%) and mixed breed (N=2; 15%). The MMVD stage B2 group (N=12; 24%) consisted of four male and eight female dogs between six (Min) and 16 (Max) years, with

a mean of 10 (±3) and a mode value of 12 (Table 1). The youngest dogs diagnosed with MMVD stage B2 were a female CKCS and a male German Spitz, both six-year-old. The oldest was a 16-year-old female Poodle. CKCS was the most represented breed in the MMVD stage B2 group (N=6; 50%). Finally, the MMVD stage C group (N=13; 26%) consisted of eight male and five female dogs between six (Min) and 16 (Max) years of age with an average of 11 (±3) and a mode value of 11 (Table 1). The youngest was a six-year-old male Yorkshire terrier and the oldest was a 16-year-old female of mixed breed. The three most frequently included breeds were CKCS (n=5; 38%), mixed breed (N=2; 15%) and Yorkshire terrier (N=2; 15%).

Several selected echocardiographic parameters measured at initial examination and cardiac imaging in dogs with different stages of MMVD are presented in Table 1. For dogs diagnosed with the MMVD stage B2, average and median value of left-atrium-to-aorta ratio (LA/Ao) was > 1.6 and left ventricular internal dimension in diastole (LVIDDn) normalized to dogs body weight was > 1.7. Furthermore, statistical difference was highly significant both for LVIDDn (Kruskal-Wallis test, $P < 0.0001$) and LA/Ao (Kruskal-Wallis test, $P < 0.0001$). For these two echocardiographic parameters measured values were statistically significantly higher with the progression of MMVD disease. Although average values for fractional shortening (FS) and mitral valve E and A velocity (MV E/A) increase with disease progression, differences were not statistically significant.

Dogs defined as having a B1 stage MMVD had a characteristic heart murmur of mitral regurgitation together with the presence of echocardiographic changes typical of myxomatous valve degeneration, but without cardiac remodelling and absence of clinical signs that could be attributable to heart failure or other diseases. Apart from the finding of a heart murmur and typical echocardiographic changes they were apparently healthy. MMVD stage B2 dogs were defined by the finding of a typical heart murmur, presence of echocardiographic evidence of MMVD but with cardiac remodelling, and absence of current or previously reported sings of

heart failure, primarily pulmonary oedema on thoracic radiographs. The stage C dogs had a typical heart murmur with maximal intensity over the mitral valve area, had echocardiographic evidence of advanced MMVD (characteristic lesions of the mitral valve apparatus) together with mitral valve regurgitation and moderate to severe left atrial enlargement, and demonstrated current or prior radiographic evidence of cardiogenic pulmonary oedema.

The MMVD development and progression in dogs is not completely elucidated. Moreover, the MMVD in dogs has relatively unpredictable progression. In larger dogs, a faster progression with more severe cardiomegaly, apparent cardiac dysfunction and CHF may be expected. For small and medium-sized dogs, which are more frequently affected, the progression is usually slower; still, an early onset of disease, rapid progression and fatal outcome can be expected for these dogs to. In our study, the waste majority of dogs diagnosed with MMVD were of small or medium-sized breed (Table 1). This is in agreement with previously mentioned frequency based on a breed-size. From presented data (Table 1) we were unable to discuss the effect of breed size on the MMVD onset and advancement as all diseased dogs were of small or medium size. The lack of inclusion of larger dogs in the study could also be ascribed to the fact that progression of disease in large breeds could be rapid and with fatal outcome. Consequently, for these dogs on-time diagnosis was not conducted. However, it is more likely that progression in larger breeds is very slow with no visible signs and no or limited impact on dogs' life. Therefore, such dogs may die of other age-associated diseases, while MMVD remains hidden and undiagnosed.

Novel research studies suggest that MMVD progression and morbidity are directly related with the grade of mitral insufficiency and volume overload while age, severity of left atrial enlargement and heart rate show moderate potential for prediction of disease progression and outcome. Experimental studies suggest that some pure breed dogs, for instance, Cavalier King Charles Spaniel and Dachshund, show increased risk for an early onset and rapid progression

of MMVD. Moreover, male dogs seem to be more frequently affected with more severe progression and increased cases of mortality rates. Due to intentional balanced gender distribution in our study (Table 1) we were unable to make similar conclusions. However, increased frequency of appearance of pedigree breeds (CKCS and Maltese dog) in the MMVD groups was observed, specifically for symptomatic stages, that is, the MMVD stages B2 and C (Table 1). Based on published data and clinical experience, MMVD usually has a long asymptomatic period, due to slow progressive degeneration that might with time result in eccentric hypertrophy of left side of the heart. Cases of MMVD with long progression may be considered as a chronic disease, which either becomes symptomatic and visible to dog owners or is diagnosed during routine annual clinical examination. In both instances, dogs routinely diagnosed with MMVD are usually more than 10 years old. In line, patients included in this study were mostly older than 10 years (66%). On the other side, when polygenic inheritance is present, as in case of CKCS breed and some others, an earlier MMVD onset is very likely. This can also be observed from presented data (Table 1), for instance, 54% of dogs diagnosed with MMVD were of CKCS breed and below 10 years of age. Moreover, the youngest diagnosed dog (age 4) was also of CKCS breed. Therefore, the MMVD for dogs of CKCS breed presents a serious welfare problem. In addition, in our study an increased number of dogs with MMVD (regardless of stage) were small/medium sized dogs of mixed breed (Table 1).

The MMVD changes mechanical capacity of diseased valve; therefore, typical heart murmur, mitral valve prolapse and regurgitation are common discoveries during clinical examination of patients. A characteristic systolic heart murmur of moderate to high severity with point of maximal intensity over the mitral valve area, typical echocardiographic changes of MMVD (leaflet thickening and prolapse, regurgitation) were also observed for diseased dogs included in the study. Exposure to constant and progressive regurgitation amplifies deformation of mitral valve structure, leads towards increased cardiac work, ventricular remodelling and, finally,

ventricular dysfunction with systemic effects and various complications. In accordance with data presented for our patients (Table 1), the prevalence of canine MMVD is higher in small- and medium-sized breeds and crossbreeds (< 20 kg), while gender, age and heredity seem to be contributing factors to early and increased incidence and rapid progression of disease. Although care was taken to match demographics between the groups of dogs diagnosed with MMVD and the control group, healthy/control dogs were statistically significantly younger (P=0.0255) from dogs in the MMVD stages B1, B2 and C. This is presumably a consequence of the nature of MMVD since this disease is mostly diagnosed in older dogs.

Section S2. Supplement on serum biochemical analysis

The following biochemical parameters were measured in serum: albumin, alkaline phosphatase (ALP), alanine aminotransferase (ALT), aspartate aminotransferase (AST), creatine kinase (CK), C-reactive protein (CRP), gamma-glutamyl transferase (GGT), glucose, cholesterol, creatinine, lactate dehydrogenase (LDH), triglycerides, bilirubin, proteins, urea nitrogen, calcium, chlorine, phosphorus, potassium, magnesium, and sodium. Serum urea nitrogen to creatinine ratio was calculated. Serum chloride concentrations [Cl^-] were reported as corrected serum chloride [corr. Cl^-] for serum sodium concentration [Na^+] using the formula (148/[Na^+]) x [Cl^-] in which 148 is the middle value of reference range (5).

As indicated in Table S1, activities of ALP and AST as well as concentrations of urea nitrogen, corrected chloride and phosphorus, were statistically significantly different at P=0.05 between healthy/control and different MMVD stages. Specifically, the ALP activity in serum was statistically significantly higher in MMVD stages B1, B2 and C compared to healthy/control group (Kruskal-Wallis test, P=0.0449). The average values of ALP activities for MMVD stage B1, B2 and C were above the maximal value of reference interval. In addition, the AST activity

in serum was statistically significantly lower in MMVD stage C dogs compared to the MMVD stage B1 and healthy/control group (Kruskal-Wallis test, P=0.0426). On the other hand, the serum CK activity was statistically significantly higher for MMVD stage B1 compared to MMVD stages B2 and C (Kruskal-Wallis test, P=0.0321). Average CK and AST activities were not outside the reference range. Finally, the chloride concentration in serum was statistically significantly lower for the MMVD stage C dogs separately compared to the MMVD stages B1, B2 and healthy/control group (Kruskal-Wallis test, P=0.0544). Average chloride concentrations were slightly above the maximal value of the reference interval for healthy/control group (4 mmol/L above the upper border of reference range), MMVD stage B1 (2 mmol/L above the upper border of reference range) and MMVD stage B2 (2 mmol/L above the upper border of reference range).

Furthermore, differences of phosphorus concentrations between healthy and different MMVD stages were statistically highly different (Kruskal-Wallis test, P=0.0025), while differences of urea nitrogen concentrations and urea to creatinine ratio were statistically very highly significant (Kruskal-Wallis test, P=0.0001). Specifically, the phosphorus concentration in serum of dogs with diagnosed MMVD stage C was statistically highly significantly higher when compared to the diagnosed MMVD stages B1 and B2 as well as healthy/control group. All average concentrations of phosphorus were inside the reference range. The concentration of urea nitrogen in serum was very highly significantly higher in MMVD stage C dogs compared to the MMVD stages B1 and B2 and and in MMVD stage B1 compared to healthy/control group. The average concentrations of urea nitrogen for MMVD groups B1 and C were above the normal reference range. The urea nitrogen/creatinine concentration ratio was very highly significantly higher in MMVD stage C compared to the MMVD stages B1 and B2 as well as to healthy/control group (Kruskal-Wallis test, P=0.0001). In addition, the urea

nitrogen/creatinine ratio was very highly significantly higher in MMVD stages B1 and B2 compared to healthy/control group.

Several mean serum parameters were modestly above the upper border of reference interval, however significant difference was not shown (Table S1). These were: mean CRP concentrations for MMVD stages B1 (6.2 mg/L above upper range) and C (8.6 mg/L above upper range), mean GGT activities in MMVD stages B1 (0.7 U/L above upper range), B2 (1.3 U/L above upper range) and C (0.2 U/L above upper range), mean triglycerides concentration of the MMVD stage B2 (0.4 mmol/L above upper range) and mean cholesterol concentrations for MMVD B1 (3 mmol/L above upper range), B2 (2.6 mmol/L above upper range), C (2.4 mmol/L above upper range) and healthy/control group (1.9 mmol/L above upper range) (Table S1).

Table S1. Serum biochemical profile across three stages of naturally occurring myxomatous mitral valve disease (MMVD) and healthy dogs.

Parameter (unit)	Reference interval* (Min-Max)	Healthy dogs (Median (Q1-Q3)	MMVD stage B1 (Median (Q1-Q3)	MMVD stage B2 (Median (Q1-Q3)	MMVD stage C (Median (Q1-Q3)
Biochemical parameters					
Albumin (g/L)	26-33	33 (31.3-34)	33 (27.8-35.5)	31 (29-33)	31 (30-35)
ALP (U/L)	20-156	30 (21.8-44.8) [b, c, d]	112.5 (26.8-248.3) [a]	151 (30-586) [a]	81 (48-338.5) [a]
ALT (U/L)	0-88	34 (17-73.5)	45.5 (24.3-74.3)	35 (23-53)	44 (28-78.5)
AST (U/L)	0-82	18 (13-26) [d]	18.5 (17-20.8) [d]	16 (13-19)	13 (11-17.5) [a, b]
CK (U/L)	0-160	76 (36.3-132.8)	112 (82.3-157.8) [c, d]	82 (49-102) [b]	59 (39-88.5) [b]
CRP (mg/L)	0-10.7	1 (1-1)	1 (1-4.3)	1 (1-1.6)	1.63 (1-29.9)
GGT (U/L)	0-6	5 (4-6.3)	6 (5-7)	5 (4-6)	6 (5-7)
Glucose (mmol/L)	3.6-6.5	5.3 (5.1-5.7)	5.2 (4.7-5.5)	5.4 (4.9-5.8)	5.2 (4.5-5.5)
Cholesterol (mmol/L)	3.5-5.1	6.8 (5.9-7.6)	6.9 (5.9-9.9)	7.3 (5-10.6)	7.2 (6-8.6)
Creatinine (µmol/L)	44-140	87.5 (70.5-94.8)	80.5 (68-115.8)	71.4 (66-92)	106.6 (89-170)
LDH (U/L)	45-233	47.5 (30.8-161)	127.5 (62.8-172.3)	59 (51-96)	90 (37.5-129)

Triglycerides (mmol/L)	0.2-1.3	0.53 (0.4-1.6)	1.01 (0.5-2)	1.15 (0.4-1.9)	0.93 (0.7-1.1)
Bilirubin (µmol/L)	1.7-8.6	1.3 (1-1.5)	1.12 (0.9-1.3)	1.07 (0.9-1.5)	1.36 (1.2-1.8)
Proteins (g/L)	55-75	68.5 (62-73.5)	65 (59.5-72.5)	68 (67-73)	70 (65.5-75)
Urea nitrogen (mmol/L)	3.3-8.3	6.25 (4.2-7.7) [b, d]	8.4 (5.3-12.9) [a, d]	7.8 (6.5-8.5) [d]	17.3 (12.5-23.7) [a, b, c]
Calcium (mmol/L)	2.1-3.1	2.5 (2.4-2.6)	2.6 (2.4-2.7)	2.5 (2.4-2.7)	2.6 (2.4-2.8)
Corr. chloride (mmol/L)	90-110	115 (112-116) [d]	111 (109.1-112.5) [d]	112 (110-115) [d]	106 (102.9-109.3) [a, b, c]
Phosphorus (mmol/L)	0.7-2.1	1.5 (1.2-1.6) [d]	1.3 (1.2-1.4) [d]	1.4 (1.0-1.7) [d]	1.9 (1.7-2.2) [a, b, c]
Potassium (mmol/L)	3.6-5.8	4.6 (4.5-4.9)	4.9 (4.7-5.6)	4.9 (4.6-5.2)	4.6 (4.3-5.3)
Magnesium (mmol/L)	0.8-1.1	0.8 (0.7-0.8)	0.8 (0.8-0.9)	0.8 (0.7-0.8)	0.7 (0.7-0.9)
Sodium (mmol/L)	140-155	152 (148-157)	150 (147.3-154.3)	150 (148-154)	153 (152-157.5)
Urea nitrogen/ creatinine	-	69.56 (63.13-82.37) [b, c, d]	97.17 (70.93-135.1) [a, d]	100 (86.95-122.45) [a, d]	144.5 (121.2-162.57) [a, b, c]

Min=minimum; Max=maximum; Q1= first quartile; Q3= third quartile; ALP=alkaline phosphatase, ALT=alanine aminotransferase, AST=aspartate aminotransferase, CK=creatine kinase, CRP=C-reactive protein, GGT=gamma-glutamyl transferase, LDH=lactate dehydrogenase

*Reference interval values of the Laboratory of the Internal Diseases Clinic, Faculty of Veterinary Medicine at University of Zagreb

[a] statistically significantly different from control; [b] statistically significantly different from the MMVD stage B1; [c] statistically significantly different from the MMVD stage B2; [d] statistically significantly different from the MMVD stage C

Considerable overlap in the data between MMVD stages and healthy/control group can be observed, which is presumably a consequence of the nature of MMVD, that is, due to considerable variability of disease progression between patients. Nevertheless, two serum parameters were significantly different between at least two MMVD stages and the healthy/control group. These parameters were ALP and urea nitrogen/creatinine ratio, both ALP and urea/creatinine ratio were higher in diseased dogs. Furthermore, six serum parameters were significantly different between at least two MMVD stages. These parameters were CK activities and corrected chloride, urea nitrogen and phosphorus concentrations and urea nitrogen/creatinine ratio. Although, mentioned serum biochemical parameters were significantly different with disease advancement and/or compared to the healthy/control group, only average ALP activity, corrected chloride, urea nitrogen concentrations were above the reference ranges. The average ALP activity was approximately 6 times higher in MMVD stage B1, 10 times higher in MMVD stage B2 and approximately 5.5 times higher in MMVD stage C compared to healthy/control group (Table S1). Although considerable overlap was observed in measured ALP activities between various degrees of MMVD severity, a significant difference in ALP activity between MMVD stages and healthy/control group was shown (Table S1.). Increasing ALP activities observed with disease progression could potentially be a consequence of subclinical liver congestion linked to left ventricular diastolic dysfunction (6). AST and GGT activities were not significantly increased in disease compared to healthy/control group, although mean GGT activities for MMVD stages B1, B2 and C were above the reference range. To the contrary, AST activity was significantly lower in MMVD stage C compared to healthy/control group and MMVD stage B1 (Table S1). In addition, ALT activities were not significantly different, nor were measured ALT values above the upper limit of the reference range (Table S1). Results suggest that severe liver injury may potentially be excluded in these cases.

ALP is a widespread glycoprotein produced by different tissues in dog's body and is the most reported serum marker. For example, the highest activity of tissue-nonspecific ALP (TNALP) was found in bone, liver, kidneys and small intestine. Increased serum TNALP activity in dogs may point at a longer list of diseases and disorders. In this case, ALP may possibly be leaking into the blood circulation due to increased cell permeability or damaged valvular tissue, and is, therefore, not of liver origin. The probable source of ALP may be cells of mitral valve system, which go through extensive structural/cellular changes, that is, chronic valvular fibrosis. Recent biopsy studies on human and murine left ventricle (LV) revealed advanced regional LV fibrosis in the peripapillary myocardium, which developed already in subclinical phase, before evident alterations in cardiac function and because of amplified chordal tension from a prolapsing valve. Structural and cellular changes within mitral valve system could explain higher serum ALP activities early on during MMVD development in stage B1 (Table S1). In addition, regionalized LV fibrosis correlated with increased detection of macrophages and myofibroblasts in fibrotic areas (7). Similar research on canine model are scarce.

Considerable data variability inside experimental groups can be observed for ALP activities measured separately for MMVD B1, B2 and C (Table S1). Variability can potentially be ascribed to differences in breed and demographic parameters inside each evaluated group (Table 1) as well as to different levels of disease progression, that is, progression of regionalized LV fibrosis, inside each experimental group. Significantly higher serum ALP activities were previously reported in dogs with moderate to severe valvular regurgitation and diagnosed chronic valve disease (4,8) that is in line with our findings (Table S1). In human medicine higher serum ALP activity is associated with vascular calcification, atherosclerotic disease, and an increased risk of cardiovascular events (9). Although, ALP looks like a promising marker for canine MMVD, or at least a marker for cardiovascular illness, one must be careful when making such conclusions. For instance, a natural increase in ALP activity in

dogs was observed for specific population, such as Scottish terriers and elderly dogs (10). In our opinion, additional research on circulating TNALP isoforms is needed before further evaluation of ALP clinical potential for canine MMVD.

Next to liver (CK-BB), CK isoenzymes are mostly expressed in skeletal muscles (CK-MM) and heart (CK-MB), that is, organs with high-energy requirements. The plasma concentration is usually higher in young dogs than in adults, and various muscle diseases are associated with increased values of CK in plasma (11). Significantly, increased CK activity in serum of dogs was shown for MMVD stage B1 compared to MMVD stages B2 and C though average CK values for MMVD stage B1 were not above the reference range (Table S1). CK is predominately intracellular enzyme that catalyses the reversible phosphorylation of creatine into creatine phosphate and ADP. Increased serum CK activities in subclinical phase of MMVD could potentially indicate at recent disorder within the heart cell integrity. Considerable variability was observed for CK activity, especially measured for MMVD B1 and C (Table S1). This could in theory be due to different rates of organism adjustment to valve remodelling and regurgitation and due to inclusion of different breeds and dogs of different age within one experimental group. Increase of CK activity is not specific to muscle stress or injury, also increased exercise can result with increased CK values. Before further conclusions, quantification of CK isoenzymes, particularly CK-MB, with disease progression would be of help. Previous studies report increase of CK-MB in MMVD with cardiac thrill compared to control group (12).

Hypochloremia was previously associated with CHF in dogs (13). Moreover, low serum chloride concentrations (<103.5 mmol/L) in MMVD stage D facilitate diagnosis of refractory heart failure compared to controlled cases of CHF in MMVD stage C dogs (14). In our study, corrected serum chloride concentration was significantly lower in MMVD stage C compared to MMVD stages B1, B2 and healthy/control group. Mean chloride concentration for MMVD

stage C was 106 ± 4.1 mmol/L), although still in the reference range of the laboratory, for some dogs corrected chloride concentrations below 103.5 mmol/L were measured. Assessment of serum electrolyte concentrations in treatment of stage C dogs is highly recommended, before and especially after initiating furosemide, enalapril or benazepril (15). Electrolyte abnormalities are known and common in patients with CHF, including dogs (14). For instance, in human medicine hyponatremia in CHF may fasten the decision of heart transplant (16). Hypochloremia in canine CHF may be a result of reduced appetite, vomiting and fluid/electrolyte accumulation in the lungs, which was observed for stage C cases in this study. However, it is not yet clear if hypochloremia has a direct pathological effect in MMVD. In addition, when discussing our results (Table S1) we must take into account that average corrected chloride concentrations measured for MMVD stage B1 and B2 as well as healthy/control group were slightly (4-6 mmol/L) above the reference range. Concentrations of other measured electrolytes (sodium, calcium, potassium, phosphorus, and magnesium) were all inside the reference range (Table S1). Previously an increase in serum sodium concentration in MMVD stage C was reported compared to preclinical stages (14). Significantly higher phosphorus concentration was observed for MMVD stage C separately compared to MMVD stages B1, B2 and healthy/control group. High serum phosphate concentrations were frequently observed in patients with chronic kidney failure, but also with cardiac hypertrophy (17). Interestingly, higher serum phosphate levels that were still within the laboratory reference range, as we measured for the MMVD stage C (Table S1), were earlier associated with vascular and valvular calcification in human population (18). Recent studies indicate that problem in maintenance of phosphate homeostasis may be associated with increased inflammation rate and, subsequently, risk for development of cardiovascular disease (19). Increased incidence of inflammatory cells, macrophages and T cells has already been reported in human myxomatous mitral valves. To the contrary, the number of similar studies on canine MMVD is scarce, and

those studies that were finalized first suggest that inflammation is unlikely involved in pathological process of canine MMVD (20). This conclusion is primarily due to poor evidence that inflammatory cells are present in diseased canine valves.

Urea nitrogen concentrations were significantly higher in MMVD stage C separately compared to MMVD stage B1, B2 and healthy/control group. The average urea nitrogen concentration of MMVD stage C group was 3 times higher compared to healthy/control group and above the upper border of reference interval (Table S1). Moreover, the average urea nitrogen concentrations for MMVD stage B1 and C were above the reference range (Table S1). In MMVD stage B1 50% and in MMVD stage C 100% of measured data per group were above the upper border of reference range. Azotemia is a serious medical condition, which can be a side effect of dehydration, shock and high protein diet. In addition, high blood urea nitrogen is frequently found in patients suffering from reduced kidney function or kidney failure, which may also be age-related. Creatinine concentration was not significantly different between experimental groups, although considerable variability in measured creatinine concentrations can be observed for MMVD stages B1 and C. Moreover, for six out of 13 (46 %) dogs in MMVD group C serum creatinine concentrations were above the upper border of reference interval, indicating at mild renal azotemia (creatinine concentration between 125 to 250 µmol/L) with no or mild clinical signs. Significantly higher renal parameters, urea nitrogen and creatinine concentrations, were already reported in CHF dogs compared to preclinical stages (B1 and B2) (14). Secondary renal failure due to primary heart disease, also called cardiorenal syndrome, is frequently reported in dogs with MMVD stage C (21). Left-sided CHF causes reduced blood flow to kidneys, causing reduction of glomerular filtration rate and, consequently, increased concentration of urea in blood. However, also loss of large amounts of fluid from circulation, as in pulmonary oedema which was observed for our patients, may result in pre-renal elevated urea nitrogen concentrations. Serum potassium concentration was not

reduced, and sodium concentration was not increased in MMVD stage C compared to healthy/control group (Table S1). This brings us to a conclusion that renin-angiotensin-aldosterone system may still not be active in dogs diagnosed with MMVD stage C included in this study. For MMVD stage B1 experimental group, higher urea nitrogen concentrations, which are still inside the reference range, are presumably side effects of shock and kidney stress due to increased heart rate and mitral valve regurgitation. High urea concentrations were previously associated with mortality outcomes in human patients with cardiac disease risks and, more recently, urea concentration was recognized as a predictive biomarker of cardiovascular disease in healthy, elderly population (22). Similar studies on canine MMVD are still sporadic.

Section S3. Proteomics supplement

TMT proteomics with data-dependent acquisition mode of analysis enabled quantification of 629 proteins of which 307 with at least two unique peptides and FDR < 0.001 (Supplementary material B, Excel file, sheet name: 1.Proteome discoverer export). Statistical analysis revealed the names of 84 proteins ($P < 0.05$) with significantly altered abundances (Supplementary material B, Excel file, sheet name: 2.Results of statistical analysis). Furthermore, 81 protein was quantified in ≥ 86% of samples included in the study. These proteins were quantified in more than 83% of samples per group, specifically, > 84% for group B1, > 83% for group B2, > 84 for group C and > 83% for control group. In addition, 53 proteins had an FDR value less than 5%, and of those 44 proteins were classified as master and master protein candidates. The *post-hoc* analysis identified a panel of 21 master proteins ($P <0.05$, FDR < 0.05) with significantly differential abundances in serum of experimental groups (Figure 2. and Supplementary material B, Excel file, sheet name: 3.Results of *post-hoc* analysis).

The *post-hoc* showed 16 proteins with significantly altered abundances between MMVD stage B1 and control group with adiponectin B and haptoglobin as the most significantly changed (Figure 2.). Eight proteins had higher relative abundance in serum of dogs diagnosed with MMVD stage B1 compared to control group. In PANTHER search, these proteins were classified as complement component, cytokine, transfer/carrier protein, chaperone, anticoagulant, scaffold/adaptor proteins and serine protease (Figure 2). Furthermore, eight proteins were less abundant in the MMVD stage B1, compared to the control group. These proteins were classified as non-motor actin binding protein and complement component; however, most proteins were classified as protease inhibitors and metalloproteases (Figure 2).

When MMVD stage B2 and the control group were compared, 14 proteins had significantly different abundances (Figure 2). The two proteins with the highest log2FC absolute values were adiponectin B and haptoglobin. Higher relative abundance was showed for seven proteins, the same number of proteins were less abundant in the MMVD stage B2. In addition to previous PANTHER results, coagulation factor XII was characterized as serine protease and complement C3 as protease inhibitor (Figure 2).

Among MMVD stages B1 and B2, five proteins had significantly different abundances (Figure 2.). Namely, complement C7 and C-type lectin domain family 3 member B, also known under the name tetranectin, were more abundant in MMVD stage B2. The latter proteins were identified as members of complement component and intercellular signal molecule protein classes in Panther (Figure 2.). Three proteins were less abundant in serum of dogs with MMVD stage B2 and were classified as members of cytokine and serine protease protein classes in Panther (Figure 2.).

The highest number of proteins with significantly different abundances was detected between MMVD stage C and controls. Specifically, 19 proteins had different abundances (Figure 2).

The two proteins with the highest log2FC absolute values were inter-alpha-trypsin inhibitor heavy chain 4 and with haptoglobin (Figure 2). Ten proteins were more abundant in MMVD stage C. In addition to previous findings, leucine rich alpha-2-glycoprotein 1 was classified in the Panther as scaffold/adaptor protein (Figure 2). Nine proteins were less abundant in serum of dogs with MMVD stage C (Figure 2).

For the transition from asymptomatic (stage B2) to symptomatic (stage C) MMVD cases, three proteins showed significantly different abundances (Figure 2). Leucine rich alpha-2-glycoprotein 1, recognized in Panther as a member of scaffold/adaptor protein class, was more abundant in MMVD stage C. C-type lectin domain family 3 member B and inter-alpha-trypsin inhibitor heavy chain 2 were less abundant in stage C. These proteins were classified into two protein classes, first, intercellular signal molecule protein class and, second, protease inhibitor protein class (Figure 2).

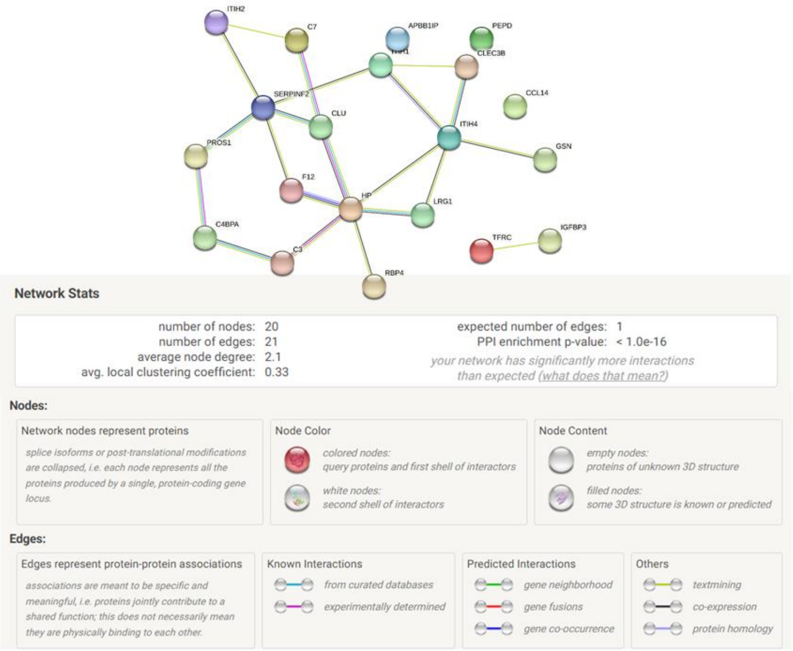

Figure S1. Protein-protein interaction networks functional enrichment presents networks for 20 significantly different serum proteins in dogs diagnosed with different stages of naturally occurring myxomatous mitral valve disease (MMVD) and healthy/control group. Networks were created in STRING (v.11.5). Search organism used: *Canis lupus familiaris*. For interactions without evidence of co-expression in *Canis lupus familiaris* putative homologs in other organisms were automatically used, mostly from *Homo sapiens*.

GSN=Actin-depolymerizing factor/Gelsolin, ITIH1=Inter-alpha-trypsin inhibitor heavy chain 1, C3=Anaphylatoxin-like domain-containing protein/complement C3, RBP4=Plasma retinol-binding protein, PROS1=Vitamin K-dependent protein S, HP=Haptoglobin, CLU=Clusterin, SERPINF2=Serpin family F member 2, LRG1=Leucine rich alpha-2-glycoprotein 1, F12=Coagulation factor XII, CCL14=C-C motif chemokine, C7=Complement C7, CLEC3B=C-type lectin domain family 3 member B/Tetranectin, TFRC=Transferrin receptor protein 1, ITIH2= Inter-alpha-trypsin inhibitor heavy chain 2, C4BPA=Complement component 4 binding protein alpha, PEPD= Peptidase D, IGFBP3=Insulin-like growth factor-binding protein 3, APBB1P=Amyloid beta precursor protein binding family B member 1, ITIH4= Inter-alpha-trypsin inhibitor heavy chain 4

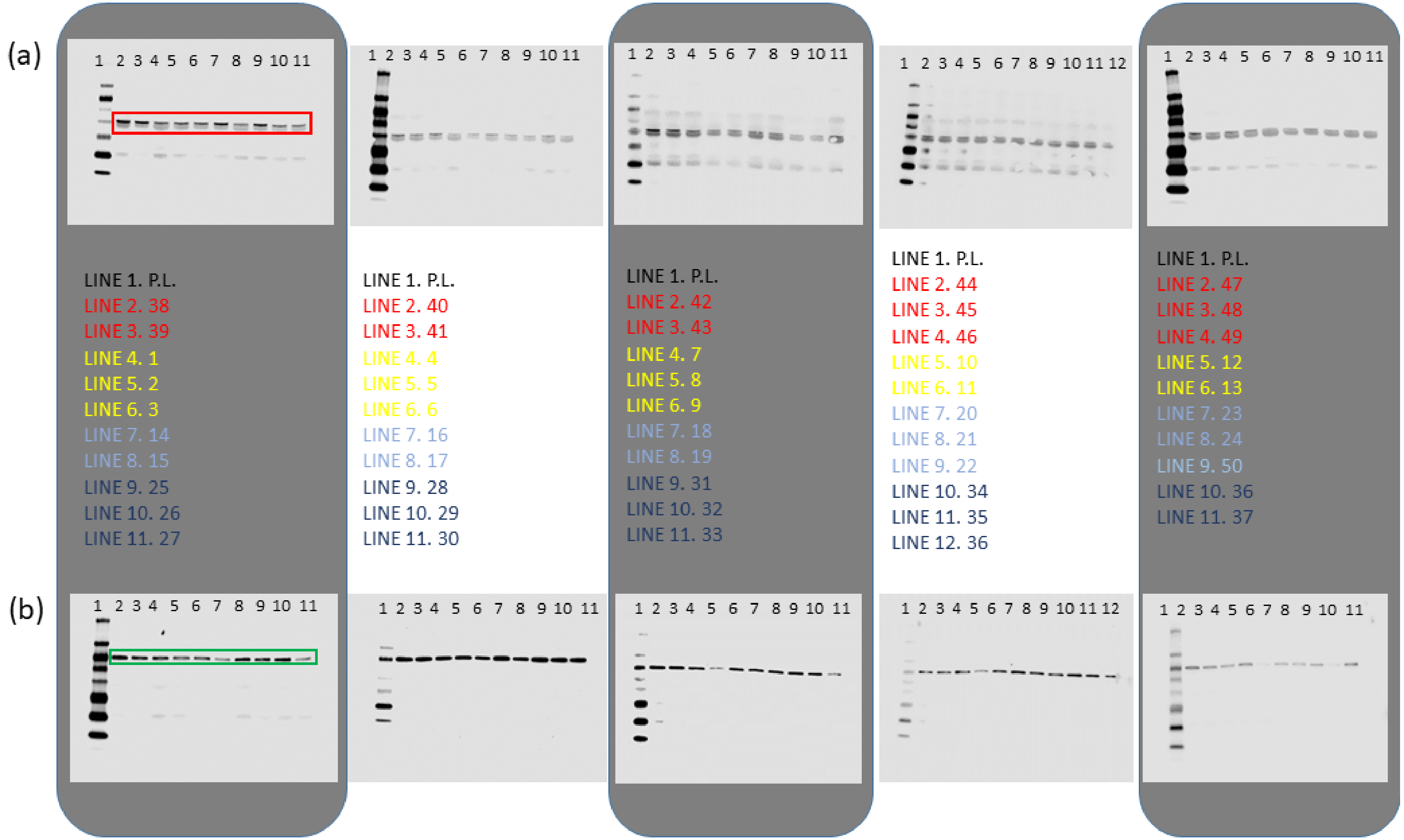

Figure S2. Western blot membranes following detection of peptidase D (PANEL A) and gelsolin (PANLE B) proteins in serum of healthy and dogs diagnosed with MMVD. Sample loading as indicated in legends, red=healthy/control group; yellow= MMVD stage B1; light blue=MMVD stage B2 and dark blue: MMVD stage C. LI-COR WesternSure® Pre-stained Chemiluminescent protein ladder (P.L.) was used as standard in lane 1 on all membranes. The representative bands shown in Figure 5c and 5d (main text) originate from unedited membranes presented here. Cropped parts are indicated with red (for figure 5c) and green (for figure 5d) rectangle.

References

1. Altschul SF, Madden TL, Schäffer AA, Zhang J, Zhang Z, Miller W, et al. Gapped BLAST and PSI-BLAST: a new generation of protein database search programs. Nucleic Acids Res. 1997;25(17):3389–402.

2. Schäffer AA, Aravind L, Madden TL, Shavirin S, Spouge JL, Wolf YI, et al. Improving the accuracy of PSI-BLAST protein database searches with composition-based statistics and other refinements. Nucleic Acids Res. 2001;29(14):2994–3005.

3. Kjelgaard-Hansen M, Jacobsen S. Assay validation and diagnostic applications of major acute-phase protein testing in companion animals. Clin Lab Med. 2011 Mar;31(1):51–70.

4. Kuleš J, Bilić P, Horvatić A, Kovačević A, Guillemin N, Ljubić BB, et al. Serum proteome profiling in canine chronic valve disease using a TMT-based quantitative proteomics approach. J Proteomics. 2020;223:103825.

5. Goggs R, Myers M, De Rosa S, Zager E, Fletcher DJ. Chloride:Sodium ratio may accurately predict corrected chloride disorders and the presence of unmeasured anions in dogs and cats. Front Vet Sci. 2017 Aug 2;4(AUG):122.

6. Ortega O, Rodriguez I, Hinostroza J, Laso N, Callejas R, Gallar P, et al. Serum Alkaline Phosphatase Levels and Left Ventricular Diastolic Dysfunction in Patients with Advanced Chronic Kidney Disease. Nephron Extra. 2011 Jan;1(1):283.

7. Morningstar JE, Gensemer C, Moore R, Fulmer D, Beck TC, Wang C, et al. Mitral Valve Prolapse Induces Regionalized Myocardial Fibrosis. J Am Heart Assoc. 2021;10(24):22332.

8. Urfer SR, Kaeberlein TL, Mailheau S, Bergman PJ, Creevy KE, Promislow DEL, et al.

Asymptomatic heart valve dysfunction in healthy middle-aged companion dogs and its implications for cardiac aging. GeroScience. 2017 Feb;39(1):43.

9. Wang Y, Liu M jie, Yang H min, Ma C yan, Jia P yu, Jia D lin, et al. Association between increased serum alkaline phosphatase and the coronary slow flow phenomenon. BMC Cardiovasc Disord. 2018;18(1):1–6.

10. Zimmerman KL, Panciera DL, Hoeschele I, Edward Monroe W, Todd SM, Werre SR, et al. Adrenocortical Challenge Response and Genomic Analyses in Scottish Terriers With Increased Alkaline Phosphate Activity. Front Vet Sci. 2018;5(OCT):231.

11. Aktas M, Auguste D, Lefebvre HP, Toutain PL, Braun JP. Creatine kinase in the dog: a review. Vet Res Commun. 1993;17(5):353–69.

12. Bakirel U, Gunes S. Value of cardiac markers in dogs with chronic mitral valve disease. Acta Vet Brno. 2009;59(2–3):223–9.

13. Adin D, Atkins C, Londoño L, Del Nero B. Correction of serum chloride concentration in dogs with congestive heart failure. J Vet Intern Med. 2021 Jan;35(1):51–7.

14. Adin D, Kurtz K, Atkins C, Papich MG, Vaden S. Role of electrolyte concentrations and renin-angiotensin-aldosterone activation in the staging of canine heart disease. J Vet Intern Med. 2020;34(1):53–64.

15. Keene BW, Atkins CE, Bonagura JD, Fox PR, Häggström J, Fuentes VL, et al. ACVIM consensus guidelines for the diagnosis and treatment of myxomatous mitral valve disease in dogs. J Vet Intern Med. 2019;33(3):1127–40.

16. Banner NR, Bonser RS, Clark AL, Clark S, Cowburn PJ, Gardner RS, et al. UK guidelines for referral and assessment of adults for heart transplantation. Heart. 2011;97(18):1520–7.

17. Christopoulou EC, Filippatos TD, Megapanou E, Elisaf MS, Liamis G. Phosphate imbalance in patients with heart failure. Heart Fail Rev. 2017;22(3):349–56.

18. Kendrick J, Kestenbaum B, Chonchol M. Phosphate and Cardiovascular Disease. Adv Chronic Kidney Dis. 2011 Mar;18(2):113.

19. Voelkl J, Egli-Spichtig D, Alesutan I, Wagner CA. Inflammation: a putative link between phosphate metabolism and cardiovascular disease. Clin Sci (Lond). 2021 Jan;135(1).

20. Aupperle H, Disatian S. Pathology, protein expression and signaling in myxomatous mitral valve degeneration: comparison of dogs and humans. J Vet Cardiol. 2012;14(1):59–71.

21. Szczepankiewicz B, Pasławska U, Siwińska N, Plens K, Pasławski R. Evaluation of the diagnostic value of the renal resistive index as a marker of the subclinical development of cardiorenal syndrome in MMVD dogs. J Renin-Angiotensin-Aldosterone Syst JRAAS. 2021;22(1).

22. Lan Q, Zheng L, Zhou X, Wu H, Buys N, Liu Z, et al. The Value of Blood Urea Nitrogen in the Prediction of Risks of Cardiovascular Disease in an Older Population. Front Cardiovasc Med. 2021;0:478.
